# Risk of severe maternal morbidity or death in relation to elevated hemoglobin A1c preconception, and in early pregnancy: A population-based cohort study

**DOI:** 10.1371/journal.pmed.1003104

**Published:** 2020-05-19

**Authors:** Alexander J. F. Davidson, Alison L. Park, Howard Berger, Kazuyoshi Aoyama, Ziv Harel, Jocelynn L. Cook, Joel G. Ray

**Affiliations:** 1 University of Toronto, Toronto, Ontario, Canada; 2 ICES, Toronto, Ontario, Canada; 3 Department of Medicine, St. Michael’s Hospital, Toronto, Ontario, Canada; 4 Department of Obstetrics and Gynaecology, St. Michael’s Hospital, Toronto, Ontario, Canada; 5 Department of Anesthesia and Pain Medicine, the Hospital for Sick Children, Toronto, Ontario, Canada; 6 Department of Obstetrics and Gynaecology, University of Ottawa, Ottawa, Ontario, Canada; 7 The Society of Obstetricians and Gynaecologists of Canada, Ottawa, Ontario, Canada; University of Edinburgh, UNITED KINGDOM

## Abstract

**Background:**

The relation between prepregnancy average glucose concentration and a woman’s risk of severe maternal morbidity (SMM) is unknown. The current study evaluated whether an elevated preconception hemoglobin A1c (A1c) is associated with SMM or maternal death among women with and without known prepregnancy diabetes mellitus (DM).

**Methods and findings:**

A population-based cohort study was completed in Ontario, Canada, where there is universal healthcare. The main cohort included 31,225 women aged 16–50 years with a hospital live birth or stillbirth from 2007 to 2015, and who had an A1c measured within 90 days before conception, including 28,075 women (90%) without known prepregnancy DM. The main outcome was SMM or maternal mortality from 23 weeks’ gestation up to 42 days postpartum. Relative risks (RRs) were generated using modified Poisson regression, adjusting for the main covariates of maternal age, multifetal pregnancy, world region of origin, and tobacco/drug dependence. The mean maternal age was 31.1 years. Overall, SMM or death arose among 682 births (2.2%). The RR of SMM or death was 1.16 (95% CI 1.14–1.19; *p* < 0.001) per 0.5% increase in A1c and 1.16 (95% CI 1.13–1.18; *p* < 0.001) after adjusting for the main covariates. The adjusted relative risk (aRR) was increased among those with (1.11, 95% CI 1.07–1.14; *p* < 0.001) and without (1.15, 95% CI 1.02–1.29; *p* < 0.001) known prepregnancy diabetes, and upon further adjusting for body mass index (BMI) (1.15, 95% CI 1.11–1.20; *p* < 0.001), or chronic hypertension and prepregnancy serum creatinine (1.11, 95% CI 1.04–1.18; *p* = 0.002). The aRR of SMM or death was 1.31 (95% CI 1.06–1.62; *p* = 0.01) in those with a preconception A1c of 5.8%–6.4%, and 2.84 (95% CI 2.31–3.49; *p* < 0.001) at an A1c > 6.4%, each relative to an A1c < 5.8%. Among those without previously recognized prepregnancy diabetes and whose A1c was >6.4%, the aRR of SMM or death was 3.25 (95% CI 1.76–6.00; *p* < 0.001). Study limitations include that selection bias may have incorporated less healthy women tested for A1c, and BMI was unknown for many women.

**Conclusions:**

Our findings indicate that women with an elevated A1c preconception may be at higher risk of SMM or death in pregnancy or postpartum, including those without known prepregnancy DM.

## Abstract 2

Joel Ray and colleagues investigate the potential risks to pregnant women with hyperglycemia.

## Author summary

**Why was this study done?:**

- Diabetes mellitus (DM) and obesity are highly prevalent, and each are associated with adverse pregnancy outcomes.
- Hemoglobin A1c (A1c) offers a convenient and representative measure of average blood glucose concentration. However, its relation to severe maternal morbidity (SMM) or death is unknown.

**What did the researchers do and find?:**

- This population-based cohort study used data from across the province of Ontario, Canada, 2007–2015. Included were 31,225 women with a live birth or stillbirth, and who had an A1c measured within 90 days before conception.
- We investigated the risk of SMM or death from 23 weeks’ gestation up to 42 days postpartum, per 0.5% absolute increase in preconception A1c.
- SMM or death arose among 682 births (2.2%). The relative risk (RR) of SMM or death was 1.16 (95% CI 1.13–1.18; *p* < 0.001) per 0.5% increase in A1c, after adjusting for maternal age, multifetal pregnancy, world region of origin, and tobacco/drug dependence.
- Among those without previously recognized prepregnancy diabetes and whose A1c was >6.4%, the adjusted relative risk (aRR) of SMM or death was 3.25 (95% CI 1.76–6.00; *p* < 0.001).

**What do these findings mean?:**

- An elevated preconception A1c is associated with a higher risk of SMM or death among women without known diabetes, and below thresholds commonly used to diagnose DM.
- These findings underscore the importance of a healthy diet and lifestyle before pregnancy, and call attention to the value of identifying women with varying degrees of hyperglycemia before pregnancy.

## Introduction

The prevalence of diabetes mellitus (DM) [1] and obesity [2] have rapidly increased worldwide. Serum glycated hemoglobin A1c (A1c), expressed as an absolute percent (e.g., 5.5%), offers a convenient and representative measure of average blood glucose control among individuals with DM [3] and of prediabetes in obese adults [4]. Prepregnancy DM and maternal obesity are each associated with a higher risk of congenital anomalies [5,6] and preterm birth [7,8].

Studies have shown that hyperglycemia adversely effects placental development [9,10], and the placenta of a diabetic mother is more likely to enter a state of oxidative stress by the first trimester, a precursor to the development of preeclampsia [11,12]. Maternal obesity is also connected with abnormal placental development [13], including a higher risk of preeclampsia [14] and severe maternal morbidity (SMM) [15]. Chronic hypertension, a condition associated with obesity and hyperglycemia [16], is also linked with a higher risk of preeclampsia [17] and SMM [18].

In mothers with preexisting DM and prediabetes, rising A1c is associated with preeclampsia [19]. However, beyond preeclampsia—a major contributor to SMM [20]—the relation between average glucose concentration in early pregnancy and a woman’s risk of SMM is unknown. SMM includes a variety of indicators that exponentially increase the risk of maternal death, and which can be assessed using population-based healthcare administrative data [20,21]. As SMM comprises about 40 indicators arising in pregnancy, during labor, or postpartum [22], many, but not all, are plausibly related to maternal obesity, DM, or average glucose concentration (S1 Fig).

Given the known relation between both of DM and obesity and adverse pregnancy outcomes, the possibility that hyperglycemia may be associated with SMM, and the widespread availability of A1c testing, the main objective of the current study was to evaluate whether elevated preconception A1c is predictive of SMM or death, including women without previously recognized preconception DM. A secondary objective was to evaluate whether any elevated A1c in the first part of pregnancy is also associated with SMM or death.

## Methods

This retrospective population-based cohort study was completed using existing datasets across the province of Ontario, where healthcare is universal. Datasets were linked using unique encoded identifiers and analyzed at the Institute for Clinical and Evaluative Sciences (ICES). All hospital live births and stillbirths in Ontario were identified in the Canadian Institute for Health Information’s Discharge Abstract Database (CIHI-DAD) and linked to the Ontario Laboratories Information System (OLIS), which captures the majority of outpatient laboratory testing in the province from March 2007 to December 2015. Gestational age in the CIHI-DAD is derived from the best clinical estimate recorded in the medical chart, largely based on ultrasound dating [23]. At least 95% of births in Ontario have an ultrasound that permits accurate pregnancy dating [24]. Other sources used were the Better Outcomes Registry and Network (BORN) database; the Ontario Diabetes Dataset; the Registered Persons Database; Statistics Canada census data; the Ontario Health Insurance Plan (OHIP) Claims Database; and the Immigration, Refugees and Citizenship Canada Permanent Resident Database. Specifics about databases and diagnosis codes are included in S1 Table. This study is reported as per the Strengthening the Reporting of Observational Studies in Epidemiology (STROBE) guideline (S1 Checklist).

### Participants

Eligible participants were all women with a live birth or stillbirth from 23 weeks’ gestation onward, occurring in an Ontario hospital. Of these women, those who underwent A1c screening between March 2007 and December 2015 formed the A1c cohort—analyzed as the main sub-cohort of women who had A1c testing preconception, as well as a secondary sub-cohort of women who had an A1c in early pregnancy (outlined below). As also explained below, in an additional analysis, the A1c cohort was contrasted with women who did not undergo A1c screening during the same period (the non-A1c cohort) to characterize any differences, including the risk of SMM or death.

Excluded from the study were women <16 years or >50 years at the time of conception; non-Ontario residents; those without a valid OHIP number or who were otherwise ineligible for OHIP; and women who gave birth or died prior to 23 weeks’ gestation (S2 Fig).

### Exposures

Within the A1c cohort, two different exposure periods were considered. The main exposure group—the preconception sub-cohort—comprised women who had an A1c from minus 90 days up to the approximate date of conception, the typical life span of a red cell [25] and a reflection of average glucose concentration [26]. If a woman in the preconception sub-cohort had more than one A1c test, the one nearest to conception was used.

Additionally, the in-pregnancy sub-cohort comprised those who had an A1c from the estimated date of conception up to 21 completed weeks’ gestation, which generally precedes the potential physiological decline in A1c seen by midpregnancy [27,28]. Among the in-pregnancy sub-cohort, the A1c test furthest from conception was used, to minimize the temporal separation of glycemic measurement and the potential onset of SMM. A woman in the preconception sub-cohort could also enter the in-pregnancy sub-cohort.

In this study, A1c was evaluated as a percent of total hemoglobin (as recommended by Diabetes Canada) [26], and tests reported to OLIS in mmol/mol were converted using the formula provided by the International Federation of Clinical Chemistry [3]. Under public health regulations in Ontario, A1c assays are held to a high standard of precision and must be certified every year by the United States National Glycohemoglobin Standardization Program (NGSP) [27]. In the US, where the American Diabetes Association (ADA) recommends that labs achieve NGSP certification, the coefficient of variation on A1c measurement has improved from approximately 5% to 6% in 2000 to less than 3.5% in 2018 [28].

### Outcomes

The primary study outcome was SMM or death arising from 23 weeks’ gestation (the beginning of newborn viability) [29] up to 42 days after the index birth (the conventional postpartum period). A secondary study outcome was SMM or death arising from the index birth up to 42 days thereafter. SMM, identified according to the definition developed by the Canadian Perinatal Surveillance System, offers a pragmatic and validated proxy for maternal “near miss” and prolonged hospital length of stay, and it can be ascertained using population-based healthcare administrative data [20,22,30]. The SMM composite comprises about 40 morbidity measures arising in pregnancy, during labor, or postpartum (see S1 Table and S1 Fig). The most common SMM indicators are intensive care unit admission, invasive ventilation, cardiac conditions, complications of obstetric surgery or procedures, and postpartum hemorrhage with blood transfusion [20,22,30].

### Covariates

Study covariates were derived from the same databases as the exposure and outcome variables and are detailed under “covariates” in S1 Table.

### Statistical analysis

Demographic and clinical variables were tabulated for preconception and early-pregnancy sub-cohorts, whose respective characteristics were compared using standardized differences, with a value >0.10 suggesting an important difference.

A plot was made of the continuous relation between preconception A1c and the unadjusted probability of SMM or death from 23 weeks’ gestation up to 42 days after birth, using modified Poisson regression [31].

The relation between preconception A1c and the outcome of SMM or death was quantified by a relative risk (RR), derived using modified Poisson regression with a robust error variance, which also accounts for correlated errors among pregnancies within the same woman (main model) [31]. A1c was expressed in increasing 0.5% absolute increments, a value that is easy to remember, and which reflects a change in A1c considered clinically important by the ADA and the United Kingdom National Institute of Health and Clinical Excellence [27]. RRs were adjusted for covariates chosen a priori, including maternal age, multifetal pregnancy, and world region of origin (including Canadian-born women)—each at the time of A1c screening—as well as tobacco/drug dependence within one year preceding the estimated date of conception.

This study used a prospective protocol (see S1 Prospective Protocol). All analyses were planned in advance of receiving data from ICES with the exception of Additional analysis 9 which was modified on peer review. Analyses were performed using SAS statistical software, version 9.4 (SAS Institute, Cary, NC).

### Additional analyses

As SMM largely occurs around the time of a birth [20], the main model was rerun, analyzing preconception A1c and the subsequent risk of SMM or death from the birth date up to 42 days thereafter (Additional analysis 1).

Obesity may be an important risk factor for maternal morbidity and death. Accordingly, the main model was restricted to a subset of women with a record of prepregnancy body mass index (BMI) in the BORN database, which was added as a covariate to the main model (Additional analysis 2). Likewise, chronic hypertension and chronic kidney disease may each confound the relation between A1c and SMM or death [20]. Accordingly, diagnosed chronic hypertension and serum creatinine [32]—each within one year preceding the estimated date of conception—were added to the main model (Additional analysis 3). Given the potential effect of anemia on A1c concentration [33], the maternal hemoglobin concentration closest to the preconception A1c screening was also added to the main model (Additional analysis 4).

The main model was then stratified a priori by factors that might influence the absolute or relative risk of a woman developing SMM, including maternal age <40 or ≥40 years, nulliparous and parous women, singleton and multifetal pregnancies, area-level income quintile, urban or rural residence, known prepregnancy DM, and prepregnancy chronic hypertension [17,18,20] (Additional analysis 5). A woman was classified as having prepregnancy DM if she was in the Ontario Diabetes Dataset at any point prior to the estimated date of conception of the index pregnancy.

Women who newly develop gestational diabetes mellitus (GDM) in pregnancy may be at higher risk of maternal morbidity or death. Accordingly, the preconception sub-cohort was further limited to women without known prepregnancy DM, and the main model was additionally adjusted for GDM (Additional analysis 6).

A1c is recommended for monitoring average glucose concentration in those with preexisting DM, as well as to screen for DM in nonpregnant adults [4,26,27]. For the latter, conventional A1c cut-points have been used to classify individuals as “normal” (<5.8%), “prediabetes” (5.8%–6.4%), and “DM” (>6.4%) [26,27]. Hence, we evaluated the risk of SMM or death comparing those whose A1c fell into either the prediabetes or the DM category, each relative to women in the normal A1c category (Additional analysis 7). This analysis was conducted among all women in the preconception sub-cohort, as well as limited to those not previously diagnosed with prepregnancy DM.

Many women undergo A1c testing once pregnant. Accordingly, we reran the main model but changed the exposure period to start at the estimated date of conception and to end at 21 completed weeks’ gestation—the in-pregnancy sub-cohort (Additional analysis 8). Among the in-pregnancy sub-cohort, the mean (95% CI) A1c concentration was plotted by increasing gestational week, and a linear trend and r^2^ (95% CI) were calculated therein. Post hoc, the gestational age at A1c testing was further added to the multivariable model in Additional analysis 9.

A woman who undergoes A1c screening may differ from one who does not. To address this point, we selected women in the non-A1c cohort who otherwise met the same criteria as those who had a preconception A1c. Baseline variables were then compared between the non-A1c cohort and the preconception sub-cohort, using standardized differences (Additional analysis 10).

Some SMM indicators may be more plausibly related to maternal average glucose concentration than others. A priori, two authors (AJFD and JGR) informally searched the literature and classified the SMM indicators into three groups: those likely, possibly, or unlikely to be related to A1c (S3 Table). Thus, the main model of preconception A1c was evaluated in relation to the likely, possibly, or unlikely outcome of SMM. If a woman had more than one SMM indicator, then a likely indicator overrode a possibly indicator, and a possibly indicator overrode an unlikely indicator (Additional analysis 11).

### Patient and public involvement

It was not appropriate or possible to involve patients or the public in the design, conduct, reporting, or dissemination of our research.

### Details of ethics approval

The use of data in this project was authorized under section 45 of Ontario’s Personal Health Information Protection Act, which does not require review by a Research Ethics Board.

## Results

There were 115,992 pregnancies in OLIS with at least one A1c measured as an outpatient within 90 days preconception, or from the estimated date of conception to 21 completed weeks’ gestation; of these, 114,896 (99.1%) comprised the entire A1c cohort (S2 Fig). Within the A1c cohort, 31,225 pregnancies (27.2%) had an A1c test within the last 90 days before the estimated date of conception, forming the preconception sub-cohort (Table 1). Among the latter, the mean maternal age was about 31 years, 43.3% were nulliparous, 99.5% resulted in a live birth, and 38.2% of births were to immigrant women. The rate of prepregnancy DM was 10.1%, chronic hypertension 12.2%, and drug dependence/tobacco use 2.9%. A hemoglobin concentration <120 g/L was observed in 10.6% of pregnancies in the preconception sub-cohort (Table 1).

**Table 1:** Characteristics of women in the A1c cohort who were tested from minus 90 days up to the estimated date of conception (the preconception sub-cohort), and those who were tested from conception up to 21 completed weeks’ gestation (the in-pregnancy sub-cohort), with standardized differences. All data are shown as a number (%) unless otherwise noted.

Characteristic	Preconception sub-cohort (*N* = 31,225)	In-pregnancy sub-cohort^a^ (*N* = 83,671)	Standardized difference
**Maternal characteristic at the time of A1c screening**			
Mean (SD) age, years	31.1 (5.1)	30.4 (5.4)	0.14
Age by group, years			
16–19	414 (1.3)	2,124 (2.5)	0.09
20–29	11,015 (35.3)	33,617 (40.2)	0.10
30–39	18,340 (58.7)	44,444 (53.1)	0.11
40–50	1,456 (4.7)	3,486 (4.2)	0.02
Median (IQR) parity	1.0 (0.0–1.0)	1.0 (0.0–1.0)	0.05
Parity, group			
Parous	11,244 (36.0)	29,951 (35.8)	0.00
Nulliparous	13,527 (43.3)	34,792 (41.6)	0.04
Unknown	≤ 5 (0.0)	6 (0.0)	0.00
Maternal world region of origin			
Canada or long-term resident	19,307 (61.8)	52,350 (62.6)	0.01
Caribbean	610 (2.0)	1,811 (2.2)	0.01
East Asia or Pacific	2389 (7.7)	6,814 (8.1)	0.02
Hispanic America	918 (2.9)	2,291 (2.7)	0.01
Middle East or North Africa	1,464 (4.7)	3,373 (4.0)	0.03
South Asia	4,187 (13.4)	11,039 (13.2)	0.01
Sub-Saharan Africa	866 (2.8)	2,341 (2.8)	0.00
Western Nations or Europe	1,484 (4.8)	3,652 (4.4)	0.02
Residing in the lowest income quintile area	7,119 (22.8)	21,485 (25.7)	0.07
Rural or unknown residence	1,739 (5.6)	5,102 (6.1)	0.02
**Maternal conditions at any point prior to conception**			
Diagnosed DM	3,150 (10.1)	6,942 (8.3)	0.06
**Maternal conditions within 1 year prior to conception**			
Illegal drug or tobacco use	896 (2.9)	2,877 (3.4)	0.03
Chronic hypertension	3,798 (12.2)	9,892 (11.8)	0.01
Mean (SD) serum creatinine, μmol/L^b^	60.5 (10.0)	59.8 (11.5)	0.06
Mean (SD) BMI, kg/m^2^ ^c^	27.0 (7.2)	27.2 (7.6)	0.02
**Maternal conditions during the index pregnancy**			
GDM among women without known prepregnancy DM	5,276 (16.9)	14,524 (17.4)	0.01
**Characteristics of the index delivery**			
Mean (SD) gestational weeks	38.5 (2.0)	38.6 (2.0)	0.02
Multibirth	633 (2.0)	1,462 (1.7)	0.02
Stillbirth	151 (0.5)	411 (0.5)	0.00
**Hemoglobin values at the time of A1c screening**			
Mean (SD) hemoglobin concentration, g/L^d^	131.3 (10.1)	126.5 (10.3)	0.47
Hemoglobin concentration by group, g/L^d^			
<120	3,313 (10.6)	19,073 (22.8)	0.33
120–158	27,468 (88.0)	63,366 (75.7)	0.32
>158	63 (0.2)	49 (0.1)	0.04
Unknown	381 (1.2)	1,183 (1.4)	0.02
Mean (SD) A1c, percent	5.6 (0.8)	5.4 (0.6)	0.19
A1c by percent, group			
<5.8	25,012 (80.1)	71,175 (85.1)	0.13
5.8–6.4	4,250 (13.6)	9,092 (10.9)	0.08
>6.4	1,963 (6.3)	3,404 (4.1)	0.10
**From 23 weeks’ gestation up to 42 days postpartum**			
Total with SMM or death^e^	682 (2.2)	1,865 (2.2)	0.00
Mean (SD) number of SMM indicators	0.0 (0.3)	0.0 (0.3)	0.00
Total per number of SMM indicators present			
0	30,544 (97.8)	81,809 (97.8)	0.00
1	515 (1.6)	1,420 (1.7)	0.00
2	99 (0.3)	257 (0.3)	0.00
3	31 (0.1)	95 (0.1)	0.00
4	20 (0.1)	49 (0.1)	0.00
5+	16 (0.1)	41 (0.0)	0.00
**From the index birth up to 42 days postpartum**			
Total with SMM or death^f^	400 (1.3)	1,116 (1.3)	0.00

^a^Of the 31,225 women in the preconception sub-cohort, 7,383 (23.6%) were also in the in-pregnancy sub-cohort.

^b^Among 19,511 pregnancies in the preconception sub-cohort and 7,449 pregnancies in the in-pregnancy sub-cohort, which had a serum creatinine measured within 1 year before conception.

^c^Among 7,203 pregnancies in the preconception sub-cohort and 20,341 pregnancies in the in-pregnancy sub-cohort, which had a recorded prepregnancy BMI.

^d^Among 30,844 pregnancies with hemoglobin concentration measured preconception and 82,488 pregnancies with hemoglobin concentration measured in pregnancy.

^e^Among 2,547 pregnancies in the A1c cohort with SMM or death from 23 weeks’ gestation up to 42 days after the index delivery, there were 17 (0.7%) deaths.

^f^Among 1,516 pregnancies in the A1c cohort with SMM or death from birth to 42 days later, there were 15 (1.0%) deaths.

Abbreviations: A1c, hemoglobin A1c; BMI, body mass index; DM, diabetes mellitus; GDM, gestational diabetes mellitus; SMM, severe maternal morbidity

**Fig 1:**
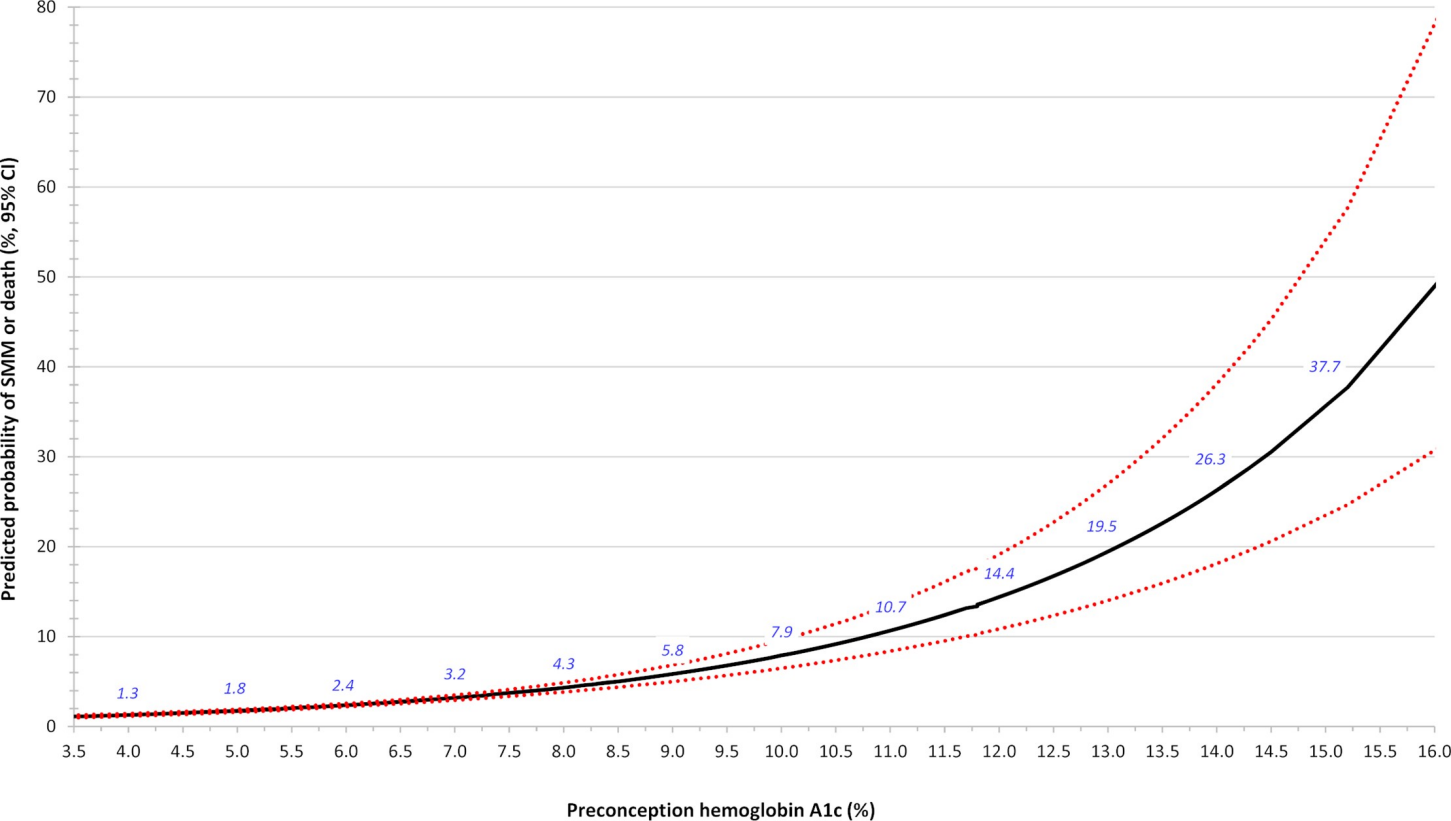
Unadjusted probability of SMM or death arising between 23 weeks’ gestation up to 42 days postpartum in relation to preconception A1c (main model). Data are presented as the absolute risk (solid black line, italicized blue values) ± lower and upper 95% confidence intervals (dashed red lines). This analysis comprises 31,225 pregnancies in the preconception sub-cohort. A1c, hemoglobin A1c; SMM, severe maternal morbidity.

In the preconception sub-cohort of 31,225 pregnancies, 682 (2.2% overall) resulted in SMM or death from 23 weeks’ gestation up to 42 days postpartum. The corresponding risk of SMM or death increased in a curvilinear manner with rising preconception A1c (Fig 1). For each 0.5% absolute rise in A1c, the unadjusted RR was 1.16 (95% CI 1.14–1.19; *p* < 0.001) and was unaffected after adjusting for the main covariates (Table 2, upper). In the preconception sub-cohort, 400 pregnancies (1.3% overall) resulted in SMM or death from the index birth up to 42 days postpartum, with an adjusted relative risk (aRR) of 1.11 (95% CI 1.07–1.16; *p* < 0.001) per 0.5% absolute rise in preconception A1c (Additional analysis 1; Table 2, lower).

**Table 2:** Risk of SMM or maternal mortality arising between 23 weeks’ gestation up to 42 days postpartum (main model, upper), as well as between the index birth up to 42 days postpartum (Additional analysis 1, lower), each in association with a 0.5% increase in preconception A1c. RRs were adjusted for maternal age and world region of origin—each at the time of the A1c test—as well as drug or tobacco dependence <1 year before conception and multifetal pregnancy. This analysis comprises 31,225 pregnancies in the preconception sub-cohort.

Timing of assessment of SMM or death	Number (overall %) with SMM or death	Unadjusted RR (95% CI)	*p*-Value	aRR (95% CI)	*p*-Value
*From 23 weeks’ gestation up to 42 days postpartum*	682 (2.2)	1.16 (1.14–1.19)	<0.001	1.16 (1.13–1.18)	<0.001
*From the index birth up to 42 days postpartum*	400 (1.3)	1.12 (1.08–1.16)	<0.001	1.11 (1.07–1.16)	<0.001

Abbreviations: aRR, adjusted relative risk; RR, relative risk; SMM, severe maternal morbidity

Upon adding prepregnancy BMI to the main model (7,203 births), the aRR of SMM or death between 23 weeks’ gestation and 42 days postpartum was 1.15 (95% CI 1.11–1.20; *p* < 0.001); adding maternal chronic hypertension and pre-pregnancy serum creatinine to the main model (19,511 births) reduced the aRR to 1.11 (95% CI 1.04–1.18; *p* = 0.002); and the addition of the periconceptional hemoglobin concentration to the main model (30,844 pregnancies) generated an aRR of 1.16 (95% CI 1.13–1.19; *p* < 0.001) (Additional analysis 2, 3, and 4, respectively).

**Fig 2:**
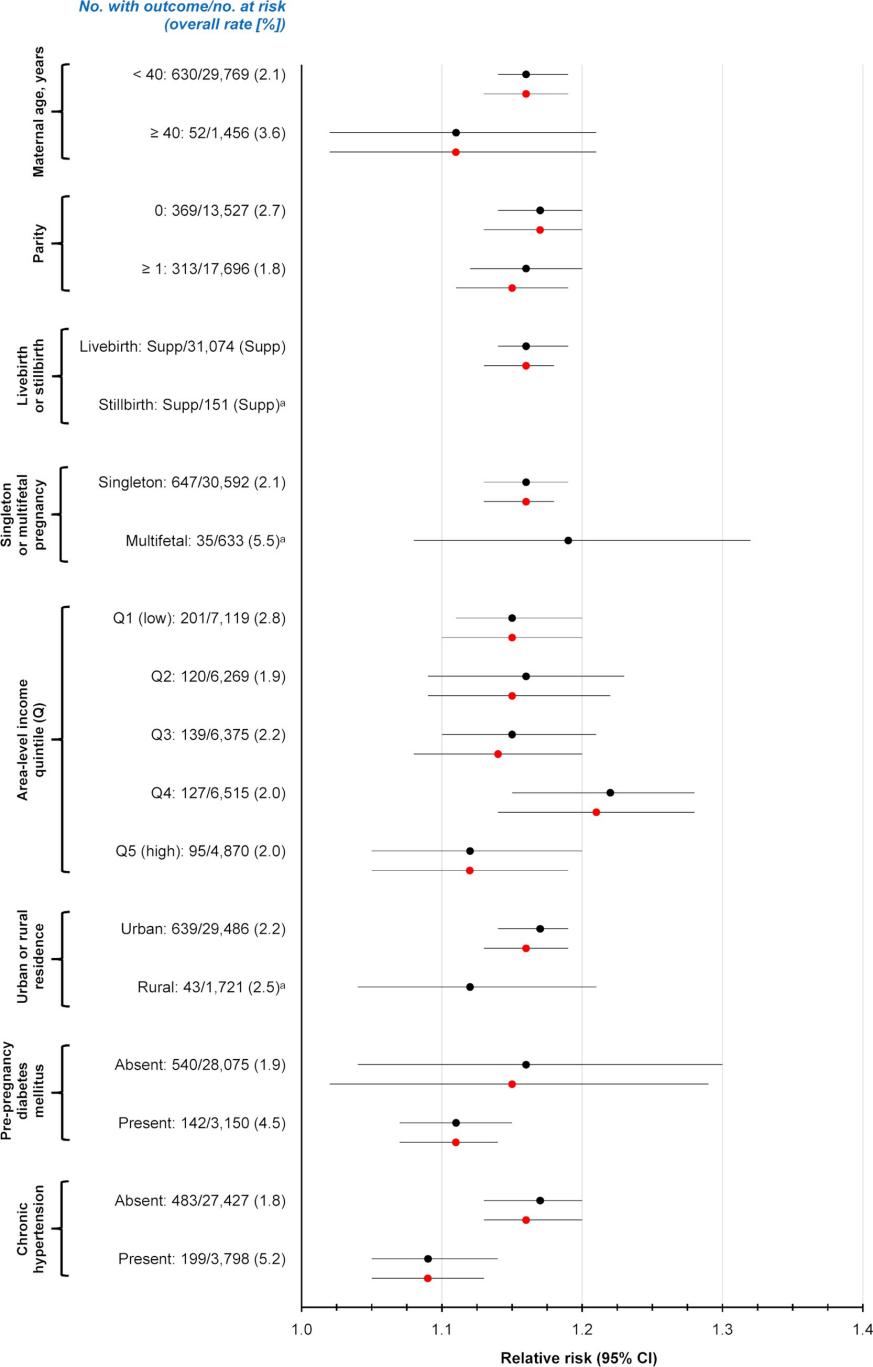
Risk of SMM or maternal mortality arising between 23 weeks’ gestation up to 42 days postpartum, associated with each 0.5% absolute increase in preconception A1c (Additional analysis 5). Shown are unadjusted (black) and adjusted (red) RRs stratified by maternal demographics and diagnosed preexisting conditions. RRs were adjusted for maternal age and world region of origin—each at the time of the A1c test—as well as drug or tobacco dependence <1 year before conception and multifetal pregnancy. This analysis comprises 31,225 pregnancies in the preconception sub-cohort. A1c, hemoglobin A1c; RR, relative risk; SMM, severe maternal morbidity.

Upon stratifying the main model by maternal demographics and preexisting conditions, all groups had an aRR of SMM or death >1.0 (Additional analysis 5, Fig 2). The rate of SMM or death was notably higher in women with prepregnancy DM (4.5%) than without prepregnancy DM (1.9%), higher with chronic hypertension (5.2%) than without (1.8%), and higher with a multifetal (5.5%) than a singleton (2.1%) pregnancy (Fig 2).

There were 28,075 women in the preconception sub-cohort without known prepregnancy DM (89.9%). Adjusting for GDM in the main model slightly attenuated the RR of SMM or death (1.12, 95% CI 1.00–1.26; *p* = 0.057) (Additional analysis 6).

Using conventional A1c cut-points, the aRR of SMM or death among the preconception sub-cohort was 1.31 (95% CI 1.06–1.62; *p* = 0.01) at a prediabetic A1c of 5.8%–6.4%, and 2.84 (95% CI 2.31–3.49; *p* < 0.001) at a diabetic A1c >6.4%, each relative to an A1c <5.8% (Additional analysis 7, Table 3). Among those without previously recognized prepregnancy diabetes, only women whose A1c was >6.4% had significantly increased risk (aRR 3.25, 95% CI 1.76–6.00; *p* < 0.001) relative to an A1c <5.8%, with SMM or death occurring in 10 out of 152 women (Table 3).

**Table 3:** Risk of SMM or death arising between 23 weeks’ gestation up to 42 days postpartum among all women (upper) and those without known prepregnancy DM (lower), each in association with preconception A1c cut-points for prediabetes (5.8% to 6.4%) and diabetes (>6.4%), relative to an A1c <5.8% (Additional analysis 7). RRs were adjusted for maternal age and world region of origin—each at the time of the A1c test—as well as drug or tobacco dependence <1 year before conception and multifetal pregnancy. This analysis comprises 31,225 pregnancies in the preconception sub-cohort, and 28,075 women without known prepregnancy diabetes therein.

	A1c category: Number (%) in that category	Number (%) with SMM or death	Unadjusted RR (95% CI)	*p*-Value	aRR (95% CI)	*p*-Value
**All women (*N* = 31,225)**	***<5*.*8%*:** 25,012 (80.1)	468 (1.9)	1.00 (referent)		1.00 (referent)	
	***5*.*8% to 6*.*4%*:** 4,250 (13.6)	104 (2.6)	1.31 (1.06–1.61)	0.01	1.31 (1.06–1.62)	0.01
	***>6*.*4%*:** 1,963 (6.3)	110 (5.6)	3.00 (2.45–3.67)	<0.001	2.84 (2.31–3.49)	<0.001
**Women without known prepregnancy DM (*N* = 28,075)**	***<5*.*8%*:** 24,406 (86.9)	453 (1.9)	1.00 (referent)		1.00 (referent)	
	***5*.*8% to 6*.*4%*:** 3,517 (12.5)	77 (2.2)	1.18 (0.93–1.50)	0.18	1.15 (0.90–1.47)	0.25
	***>6*.*4%*:** 152 (0.54)	10 (6.6)	3.54 (1.93–6.50)	<0.001	3.25 (1.76–6.00)	<0.001

Abbreviations: aRR, adjusted relative risk; A1c, hemoglobin A1c; DM, diabetes mellitus; RR, relative risk; SMM, severe maternal morbidity

Of all 114,896 births in the A1c cohort, 83,671 (72.8%) had an A1c test from conception up to 21 weeks’ gestation (the in-pregnancy sub-cohort). Except for being slightly younger in age and having a lower A1c concentration, they were similar to those in the preconception sub-cohort, including no difference in the rate of SMM or death (Table 1). Among the in-pregnancy sub-cohort, the A1c concentration dropped slightly in the first few weeks after conception, then flattened out, and rose slightly by 14 weeks’ gestation (S3 Fig). The corresponding linear trend R^2^ was 0.0008 (95% CI 0.0004–0.011; *p* < 0.001). The unadjusted RR of SMM or death was 1.17 (95% CI 1.14–1.19; *p* < 0.001) for each 0.5% increase of in-pregnancy A1c. The risk was minimally attenuated after adjusting for the main covariates (aRR 1.16, 95% CI 1.15–1.18; *p* < 0.001) (Additional analysis 8), or the post hoc further addition of gestational age at A1c testing (aRR 1.16, 95% CI 1.15–1.18; *p* < 0.001) (Additional analysis 9).

Contrasting the 31,225 pregnancies in the preconception sub-cohort with the 1,089,711 pregnancies in the non-A1c cohort, those with a preconception A1c were more likely to have South Asian ancestry, urban residence, prepregnancy DM, chronic hypertension, and lower prepregnancy BMI (Additional analysis 10, S2 Table). SMM or death was slightly more likely in the preconception sub-cohort (2.2%) than in the non-A1c cohort (1.6%)—a standardized difference of 0.04 (S2 Table).

Upon investigating preconception A1c and SMM by the a priori likelihood of such a relation (S1 Fig), the aRR of SMM was 1.19 (95% CI 1.15–1.23; *p* < 0.001) for possibly indicators and 1.17 (95% CI 1.13–1.20; *p* < 0.001) for likely indicators, with no effect for those SMM indicators deemed unlikely to be related to average glucose concentration (aRR 0.93; 95% CI 0.78–1.11; *p* = 0.417) (Additional analysis 11; S4 Table).

## Discussion

### Main findings

In this large population-based cohort study of women who had an A1c measured before conception, or in early pregnancy, an elevated A1c was associated with a higher risk of SMM or death. This was so even accounting for chronic hypertension and renal function. In an additional analysis comprising a subsample of women, BMI was not an apparent confounder of this association. While the absolute risk of SMM or death with an elevated A1c was notably higher in women with prepregnancy DM or chronic hypertension, or a multifetal pregnancy, the RR was also higher among women without known prepregnancy DM.

### Strengths and limitations

Pregnancies ending in miscarriage or induced abortion were excluded herein, as were births before 23 weeks’ gestation. This was to minimize including a pregnancy in which the date of conception might be difficult to estimate, as would the relative timing of the A1c to pregnancy. Excluding SMM or deaths beyond 42 days postpartum ensured that such events were related to pregnancy, and possibly, to early A1c levels. This research used an approach in keeping with most studies on SMM and maternal mortality, covering live births and stillbirths up to the conventional 42-day postpartum period [20–22]. As stillbirths were quite rare, stratification on the latter did not generate reliable estimates of the associated risk of SMM or death with incrementally rising A1c (Fig 2).

Most women who were pregnant during the study period did not undergo A1c testing. It is certainly possible that there was some bias for selecting women who were less healthy, thereby necessitating A1c testing. Those in the non-A1c cohort differed minimally from the preconception sub-cohort, albeit, the former had a slightly lower rate of SMM or death (S2 Table). Characteristics that notably differed between these two cohorts, such as prepregnancy BMI, DM, and chronic hypertension, were also selectively adjusted for.

While structural variants of hemoglobin, such as hemoglobin S, were known to interfere with the older A1c assays, this is not as likely with the new measurement techniques [3]. We could not account for B12 or iron deficiency, both of which prolong red cell survival and increase A1c. Although liver disease may reduce A1c, this is largely mediated by anemia, which was accounted for by controlling for hemoglobin concentration [27,33,34]. During pregnancy, A1c is thought to decline around the end of the first trimester due to greater red cell production [35] and then to increase later in pregnancy, as iron deficiency becomes more common [33]. Herein, these effects were somewhat mitigated by largely focusing on preconception A1c, while the additional analysis of in-pregnancy A1c (Additional analysis 8) may have been more profoundly influenced by such pregnancy effects.

This study had some limitations. Although the impact of confounders like obesity and renal function was considered herein, prepregnancy BMI was unknown for 77% of women, and prepregnancy serum creatinine was unknown for 38% of women. Hence, the interplay between BMI, A1c, and the risk of SMM requires further evaluation. Additionally, we did not possess details about preconception or in-pregnancy medication use, such as oral hypoglycemic agents and insulin. As about 90% of women with a preconception A1c did not have overtly diagnosed DM, the latter may not be a major shortcoming.

### Relevance and future research

Results within this cohort show that the probability of SMM rises with A1c, even below conventional A1c thresholds used to diagnose DM (Fig 1). For example, at an A1c of 5.8% (the threshold for prediabetes), the probability of SMM or death was 2.2%, while at an A1c of 6.4% (the threshold for DM), the probability was 2.6%—each considerably higher than the overall population risk of 1.7% [20]. This underscores the importance of identifying women with any degree of prepregnancy hyperglycemia, given their higher risk of SMM.

A recent population-based study of all births in Canada observed a rate of prepregnancy DM of 1.1% and obesity of 17.8% [36]. The prevalence of prepregnancy DM and obesity together was 0.48% [36]. In the US, the prevalence of obesity among nonpregnant youth is 18.5% [37], and among children with other risk factors for DM, such as obesity or a strong family history, an A1c ≥5.7% predicted the short-term risk of developing prediabetes and type 2 DM [38]. Others have shown that an A1c between 5.5% and 6.0% is associated with a 5-fold increase in the 5-year incidence of type 2 DM, compared with an A1c <5.0% [39]. Given the current and projected high rate of obesity in pregnancy, the observed association between A1c and SMM or death would suggest that A1c may be a promising biomarker of SMM in certain women. Further research is needed to validate this concept, and to determine which women may most benefit from A1c testing before and in early pregnancy, including Indigenous North Americans, who were not explicitly identified herein due to ICES policy. In addition, if screening for hyperglycemia is to be carried out in early pregnancy, the most accurate and effective test—A1c versus an oral glucose tolerance test—needs to be validated against both maternal and perinatal outcomes.

In this study, SMM indicators were bundled into those likely, possibly, and unlikely to be related to preconception A1c (S1 Fig). This novel approach suggests that not all indicators have the same association with A1c (S4 Table), which may direct future research and potential initiatives to reduce SMM.

Once a woman has been identified as vulnerable to SMM or death, there are many readily available therapies that can considerably reduce her risk of developing some of the underlying indicators of SMM. For example, a recent review in New Zealand showed that at least one third of SMM cases may be preventable [40]. Although there is no direct evidence that low-dose aspirin can prevent SMM, there is high-level support from RCTs that aspirin can reduce preeclampsia among women at risk [41–43]. Because preeclampsia is one of the major contributors to SMM [20], it is conceivable that even a mildly elevated A1c might enhance the identification of a vulnerable woman who is eligible for aspirin prophylaxis, for example. There exist several evidence-based therapies for improving glycemic control in pregnant women with prepregnancy DM, including lifestyle modification and weight loss, improved diet, and pharmaceutical treatment [44].

A1c is a commonly accepted marker of metabolic function among obese women and women with prepregnancy DM [45]. However, for women with prepregnancy DM, there is no officially recognized A1c value at which the risk of SMM rises considerably, nor is it known if A1c reduction is protective against SMM. The latter could provide another valuable reason for preconception glycemic control, in addition to the accepted prevention of congenital anomalies [46]. Hence, the potential beneficiary of this research might be both the fetus and the mother.

For women without recognized prepregnancy DM, our 90-day window used to assess preconception A1c might not have been sufficiently long enough to enable a lifestyle modification, weight reduction, and/or pharmacotherapy strategy to take effect [47].

## Conclusions

In this study, women with an elevated A1c—preconception or in early pregnancy—had an increased risk of SMM or death. Given its convenience and widespread use, A1c testing may also identify those women with preexisting DM at risk of SMM, in a manner similar to its current use in recognizing those at higher risk of fetal anomalies [5, 46], preterm birth, and preeclampsia [17,19]. As there is no current recommendation about A1c testing in nondiabetic pregnant women, especially those with obesity and/or chronic hypertension [15–18], our findings may enhance research about the benefits of A1c screening in these women.

## Supporting information

S1 STROBE Checklist STROBE checklist. STROBE, Strengthening the Reporting of Observational Studies in Epidemiology.
(DOCX)

S1 Fig Conceptual framework of the relation of prepregnancy maternal glycemia and bundles of SMM indicators (Additional analysis 11). **Green** indicates a strong theoretical relation, **yellow** indicates a possible relation, and **red** indicates an unlikely relation. For specific codes and terms, see **S3 Table**. SMM, severe maternal morbidity.
(DOCX)

S2 Fig Study flow diagram for creation of the A1c cohort from eligible deliveries that underwent outpatient A1c testing in the first 90 days before conception, or within 2 to 21 weeks’ gestation, between March 2007 and December 2015. A1c, hemoglobin A1c
(DOCX)

S3 Fig Maternal A1c concentration by gestational week, among the in-pregnancy sub-cohort of 83,671 births with an A1c test performed between conception and up to 21 weeks’ gestation. A1c, hemoglobin A1c
(DOCX)

S1 Table Variables used to define cohort entry and exclusion criteria, as well as study exposures, outcomes, adjustment, and stratification. (DOCX)

S2 Table Characteristics of the preconception A1c sub-cohort and the non-A1c cohort, with standardized differences (Additional analysis 10). All data shown are as a number (%) unless otherwise noted. A1c, hemoglobin A1c
(DOCX)

S3 Table Further information on the plausible relation between prepregnancy maternal glycemia and the various indicators of SMM^1^ (for Additional analysis 11). SMM indicators are separated into those with a **likely**, **possibly**, or **unlikely** relation to maternal average glucose concentration. Specific ICD-10-CA or CCI codes are identified, and where necessary, references are provided. CCI, Canadian Classification of Interventions; ICD-10-CA, International Classification of Diseases, 10th Revision, Canada; SMM, severe maternal morbidity.
(DOCX)

S4 Table Risk of SMM between 23 weeks’ gestation up to 42 days postpartum likely, possibly, and unlikely to be related to a 0.5% absolute increase in preconception A1c (Additional analysis 11). Green indicates a strong theoretical relation, yellow indicates a possible relation, and red indicates an unlikely relation, as outlined in **S1 Table**. RRs were adjusted for maternal age and world region of origin—each at the time of the A1c test—as well as drug or tobacco dependence <1 year before conception and multifetal pregnancy. This analysis comprises 31,225 pregnancies in the preconception sub-cohort. A1c, hemoglobin A1c; RR, relative risk; SMM, severe maternal morbidity
(DOCX)

S1 Prospective Protocol ICES Dataset Creation Plan for this project. ICES, Institute for Clinical and Evaluative Sciences.
(DOCX)

## Abbreviations

ADA: American Diabetes Association
aRR: adjusted relative risk
A1c: hemoglobin A1c
BMI: body mass index
BORN: Better Outcomes Registry and Network
CIHI-DAD: Canadian Institute for Health Information’s Discharge Abstract Database
DM: diabetes mellitus
GDM: gestational diabetes mellitus
ICES: Institute for Clinical and Evaluative Sciences
NGSP: National Glycohemoglobin Standardization Program
OHIP: Ontario Health Insurance Plan
OLIS: Ontario Laboratories Information System
RR: relative risk
SMM: severe maternal morbidity
STROBE: Strengthening the Reporting of Observational Studies in Epidemiolog
