# Supplementary material for: Risk of severe maternal morbidity or death in relation to elevated hemoglobin A1c preconception, and in early pregnancy: A population-based cohort study
Source: PLoS Med. 2020 May 19;17(5):e1003104. doi: 10.1371/journal.pmed.1003104 (PMC7236974; doi:10.1371/journal.pmed.1003104)
Supplement: S1 Fig — Green indicates a strong theoretical relation, yellow indicates a possible relation, and red indicates an unlikely relation. For specific codes and terms, see S3 Table. SMM, severe maternal morbidity. (DOCX) [file pmed.1003104.s002.docx]

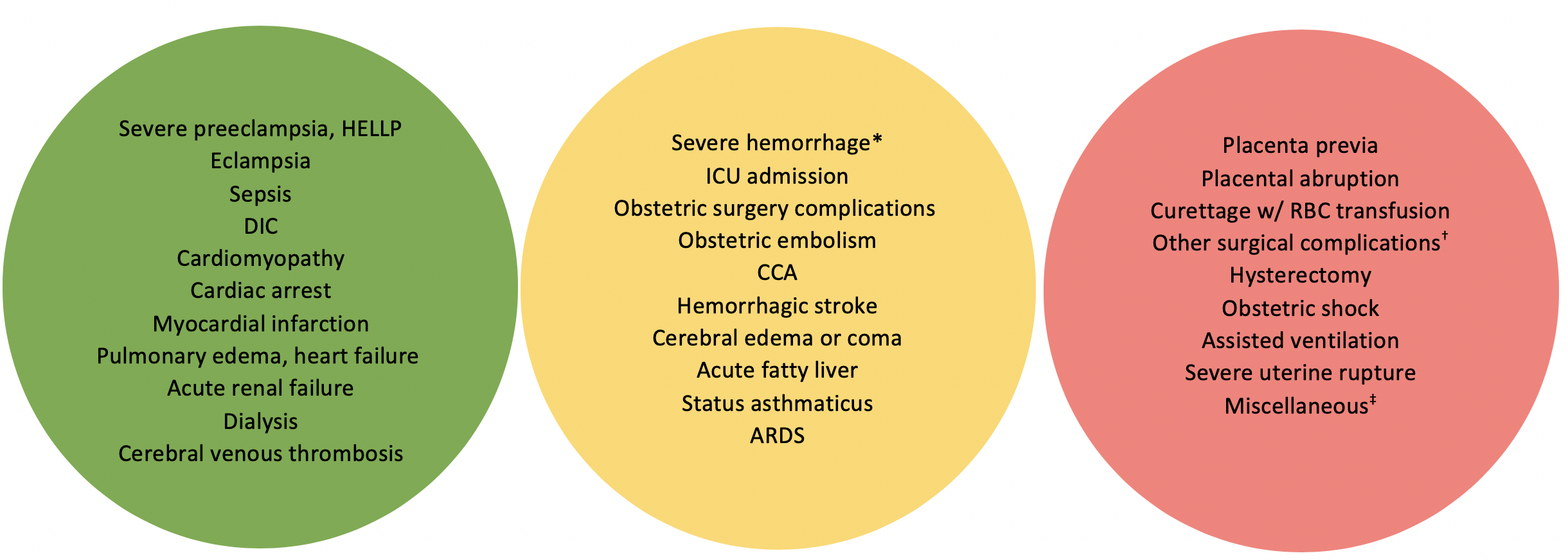
**S1 Fig. Conceptual framework of the relation of pre-pregnancy maternal glycemia and bundles of severe maternal morbidity (SMM) indicators *(additional analysis 11*).** **Green** indicates a strong theoretical relation, **yellow** indicates a possible relation, and **red** indicates an unlikely relation. For specific codes and terms, see **S3 Table**.

HELLP: Hemolysis, elevated liver enzymes, and a low platelet count

DIC: Disseminated intravascular coagulation

CCA: Cardiac complications of anaesthesia

ARDS: Adult Respiratory Distress Syndrome

RBC: Red blood cell

* Includes antepartum hemorrhage with coagulation defect; intrapartum hemorrhage with coagulation defect; intrapartum hemorrhage with RBC transfusion; and postpartum hemorrhage with RBC transfusion, procedures to the uterus or hysterectomy

† Includes evacuation of incisional hematoma with RBC transfusion; procedures to the uterus with RBC transfusion; and repair of bladder, urethra, or intestine

‡ Includes acute abdomen; correction of inverted uterus for vaginal births; sickle-cell anemia with crisis; acute psychosis; and status epilepticus
