# Supplementary material for: Risk of severe maternal morbidity or death in relation to elevated hemoglobin A1c preconception, and in early pregnancy: A population-based cohort study
Source: PLoS Med. 2020 May 19;17(5):e1003104. doi: 10.1371/journal.pmed.1003104 (PMC7236974; doi:10.1371/journal.pmed.1003104)
Supplement: S2 Fig — A1c, hemoglobin A1c (DOCX) [file pmed.1003104.s003.docx]

**S2 Fig. Study flow diagram for creation of the A1c cohort from eligible deliveries that underwent outpatient A1c testing in the first 90 days before conception, or within 2 to 21 weeks’ gestation, between March 2007 and December 2015.**

^a^Of the 31,225 deliveries in the preconception sub-cohort, 7383 (24%) were also within the in-pregnancy sub-cohort.

**115,992** eligible deliveries with an outpatient A1c measured within 90 days preconception, or from 2-21 weeks' gestation, in Ontario, Canada, from March 2007 to December 2015

**107,513** deliveries included^a^

**31,225** in the preconception A1c sub-cohort^a^

**83,671** in the in-pregnancy A1c sub-cohort

**1,096** excluded deliveries:

Invalid delivery or linked newborn hospitalization record

Missing maternal demographic data at the time of conception

Maternal age < 16 y or > 50 y at the time of conception

Non-Ontario resident at the time of conception

Maternal death at < 23 weeks' gestation

Length of gestation < 23 completed weeks
