# Supplementary material for: Risk of severe maternal morbidity or death in relation to elevated hemoglobin A1c preconception, and in early pregnancy: A population-based cohort study
Source: PLoS Med. 2020 May 19;17(5):e1003104. doi: 10.1371/journal.pmed.1003104 (PMC7236974; doi:10.1371/journal.pmed.1003104)
Supplement: S3 Fig — A1c, hemoglobin A1c (DOCX) [file pmed.1003104.s004.docx]

**
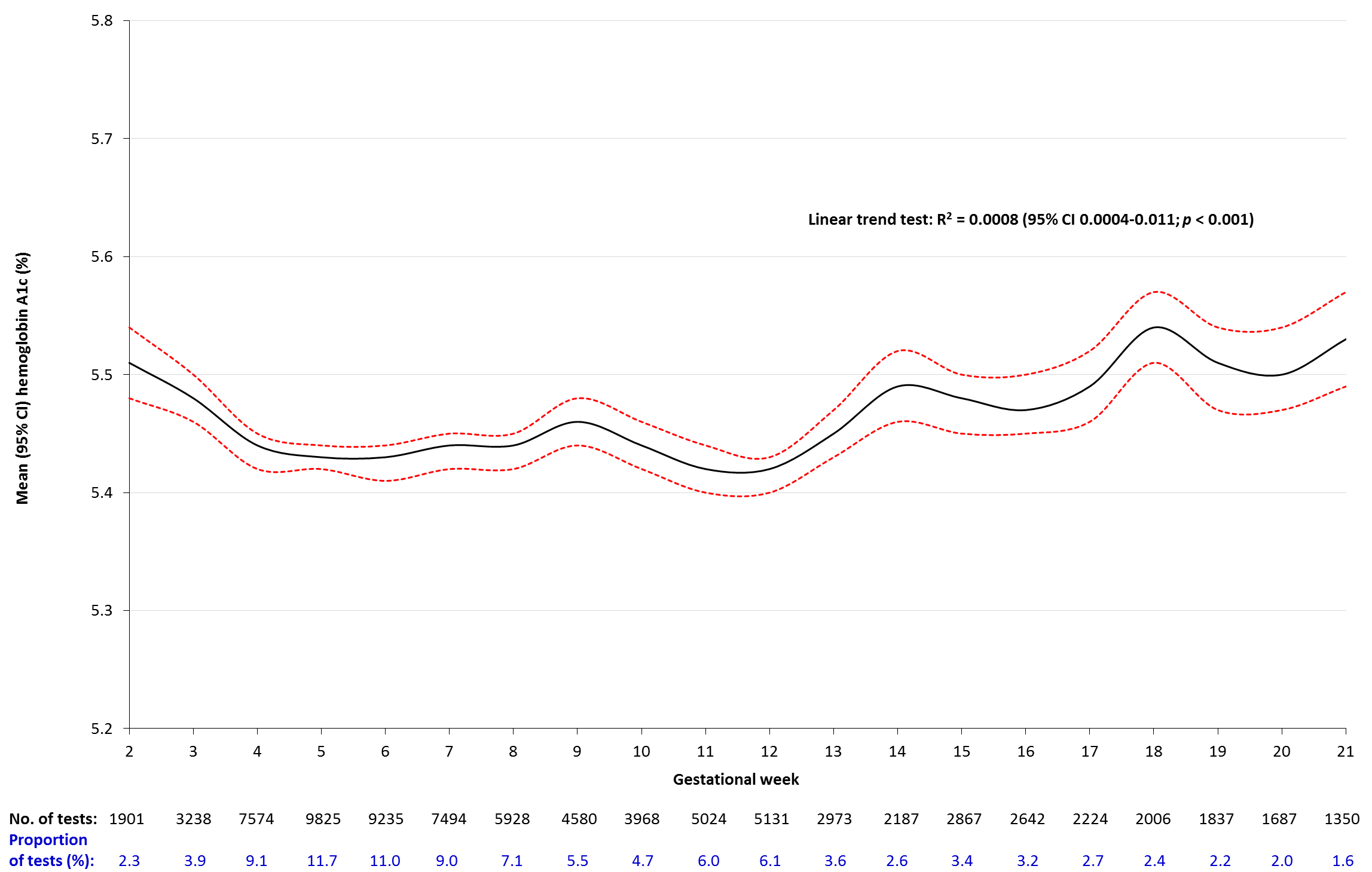
S3 Fig. Maternal hemoglobin A1c concentration by gestational week, among the in-pregnancy sub-cohort of 83,671 births with an A1c test performed between conception and up to 21 weeks’ gestation.**
