# Supplementary material for: Risk of severe maternal morbidity or death in relation to elevated hemoglobin A1c preconception, and in early pregnancy: A population-based cohort study
Source: PLoS Med. 2020 May 19;17(5):e1003104. doi: 10.1371/journal.pmed.1003104 (PMC7236974; doi:10.1371/journal.pmed.1003104)
Supplement: S1 Table — (DOCX) [file pmed.1003104.s005.docx]

**S1 Table. Variables used to define cohort entry and exclusion criteria, as well as study exposures, outcomes, adjustment, and stratification.**

| **Assessment** | **Timing** | **Disease, procedure or condition** | **ICD-10-CA or CCI codes in DAD** | **OHIP ICD-9 diagnostic codes or fee codes {or other source if in parentheses}** | **PubMed link to related validation studies for some codes** |
| --- | --- | --- | --- | --- | --- |
| *Cohort entry criteria* | January 2007 - September 2015 | Pregnant women who underwent A1c testing within 90 days before conception (preconception sub-cohort), or from 2-21 completed weeks’ gestation (in-pregnancy sub-cohort) | -- | {Ontario Laboratory Information System (OLIS) includes most outpatient laboratory information in Ontario} | -- |
| *Exclusion criteria* | At the estimated time of conception | Maternal age > 50 years or < 16 years | -- | {Registered Persons Database (RPDB) contains demographic information and encrypted healthcare numbers for all individuals eligible for OHIP} | -- |
|  | At the time of A1c testing | Non-Ontario resident, invalid OHIP number, otherwise ineligible for OHIP | -- | {RPDB } | -- |
|  | Prior to 23 weeks’ gestation | Mother died | -- | {RPDB} | -- |
| *Study exposure* | Up to 90 days before the estimated date of conception (for the main preconception sub-cohort) | Preconception hemoglobin A1c | -- | {OLIS} | -- |
|  | From the estimated date of conception up to 21 completed weeks’ gestation (for the secondary in-pregnancy sub-cohort) | In-pregnancy hemoglobin A1c | -- | {OLIS} | -- |
| *Study outcome* | From 23 weeks prior to the index birth to 42 days following birth | Severe maternal morbidity (SMM) or death | Severe preeclampsia and HELLP syndrome:  O14.1, or O14.2  Eclampsia:  O15  Cerebral venous thrombosis in pregnancy, or in the puerperium:  O22.5, or O87.3  Acute fatty liver with red blood cell (RBC) transfusion or plasma transfusion:  O26.6 + (CIHI BTREDBC = 1 or CIHI BTPLASMA = 1)  Pulmonary, cardiac, and CNS complications of anaesthesia during pregnancy, the puerperium, or labour and delivery:  O29.0, O29.1, O29.2, O89.0, O89.1, O89.2, O74.0, O74.1, O74.2 or O74.3  Placenta previa with hemorrhage with RBC transfusion:  O44.1 + CIHI BTREDBC = 1  Placental abruption with coagulation defect:  O45.0  Antepartum hemorrhage with coagulation defect:  O46.0    Intrapartum hemorrhage with coagulation defect:  O67.0  Intrapartum hemorrhage with RBC transfusion:  O67 + CIHI BTREDBC = 1  Rupture of the uterus with RBC transfusion, procedures to the uterus or hysterectomy:  (O71.0 or O71.1) + any of the following:   - CIHI BTREDBC = 1, or - (1.RM.13, 1.KT.51, 5.PC.91.LA or 5.PC.91.HV) + CIHI BTREDBC = 1, or - (5.MD.60.RC, 5.MD.60.RD, 5.MD.60.KE, 5.MD.60.CB or 1.RM.89.LA**^a^**), or - 1.RM.87.LA-GX   **^a^ NOTE**: **1.RM.89.LA** is included only if codes 1.PL.74, 1.RS.74 or 1.RS.80 are NOT also present  Postpartum hemorrhage with RBC transfusion, procedures to the uterus or hysterectomy:  O72 + any of the following:   - BTREDBC = 1, or - (1.RM.13, 1.KT.51, 5.PC.91.LA or 5.PC.91.HV) + BTREDBC = 1, or - (5.MD.60.RC, 5.MD.60.RD, 5.MD.60.KE, 5.MD.60.CB or 1.RM.89.LA**^b^**), or - 1.RM.87.LA-GX   **^b^ NOTE**: **1.RM.89.LA** is included only if codes 1.PL.74, 1.RS.74 or 1.RS.80 are NOT also present  Cardiac conditions:  O74.2, O89.1, O90.3, I21, I22, I42, I43, I46, I49.0, I50, J81, 1.HZ.09 or 1.HZ.30  Obstetric shock :  O75.1, R57, T80.5 or T88.6  Septicemia during labour:  O75.3  Complications of obstetric surgery and procedures:  O75.4  Puerperal sepsis:  O85  Obstetric embolism:  O88  Acute renal failure:  O90.4, N17, N19 or N99.0  Death, obstetric, cause unspecified:  O95  Death, obstetric, after 42 days but 1 year after delivery:  O96  Death from sequelae of direct obstetric causes:  O97  Disseminated intravascular coagulation:  D65  Sickle cell anemia with crisis:  D57.0  Acute psychosis:  F53.1 or F23  Status epilepticus:  G41  Cerebral edema or coma:  G93.6 or R40.2  Cerebrovascular diseases: subarachnoid and intracranial hemorrhage, cerebral infarction, stroke:  I60, I61, I62, I63 or I64  Status asthmaticus:  J45.01, J45.11, J45.81 or J45.91  Adult respiratory distress syndrome:  J80  Acute abdomen:  K35, K37, K65, N73.3 or N73.5  Hepatic failure:  K71 or K72  Sudden death, death from unspecified cause:  R96, R97, R98 or R99  Assisted ventilation through endotracheal tube:  1.GZ.31.CA-ND  Assisted ventilation through tracheostomy:  1.GZ.31.CR-ND  Hysterectomy:  5.MD.60.RC, 5.MD.60.RD, 5.MD.60.KE, 5.MD.60.CB, 1.RM.89.LA (exclude if 1.PL.74, 1.RS.74 or 1.RS.80 code also present), 1.RM.87.LA-GX  Dialysis:  1.PZ.21  Evacuation of incisional hematoma with RBC transfusion:  5.PC.73.JS + CIHI BTREDBC = 1  Repair of bladder, urethra, or intestine:  5.PC.80.JR, 1.NK.80, 1.NM.80  Procedures to the uterus/pelvic vessels with RBC transfusion:  (1.RM.13, 1.KT.51, 5.PC.91.LA, 5.PC.91.HV) + CIHI BTREDBC = 1  Surgical or manual correction of inverted uterus for vaginal births only:  5.PC.91.HQ or 5.PC.91.HP, restricted to vaginal births (i.e., absence of caesarean 5.MD.60)  Reclosure of caesarean wound with RBC transfusion:  (5.PC.80.JM, 5.PC.80.JH) + CIHI BTREDBC = 1  Curettage with RBC transfusion:  (5.PC.91.GA, 5.PC.91.GC, 5.PC.91.GD) + CIHI BTREDBC = 1  Maternal ICU admission:  SCU in (‘10’, ’20’, ’25’, ’30’, ’35’, ’40’,’45’,’60’,’80’) | All cause death in {RPDB} | -- |
| *Covariates* | Within one year preceding the estimated date of conception | Tobacco or drug dependence | 291, 292, 2940, 303, 304, 305, 648.3, 649.0, 6555, 980 [F10-F19, F55, G312, O354, O355, T51, T652, Z720, Z721, Z722] | 291, 292, 303, 304, 305 | -- |
|  | Same | Chronic hypertension | 401, 405, 642.0-642.2, 642.7 [I10, I15, O10, O11] | 401 | <https://www.ncbi.nlm.nih.gov/pubmed/19858407> |
|  | Same | Serum creatinine concentration | -- | {OLIS} | <https://jamanetwork.com/journals/jama/fullarticle/2720718> |
|  | At the estimated date of conception | Body mass index^a^ | -- | {BORN-Niday (April 2006-March 2012) & BORN-BIS (April 2012-March 2014)} | -- |
|  | Same | Maternal age | -- | {RPDB} | -- |
|  | Same | World region of origin | -- | {IRCC Permanent Resident Database} | -- |
|  | Same | Multifetal pregnancy | M_MULTIBIRTH=’T’ or B_MULTIBIRTH=’T’ in MOMBABY | -- |  |
|  | Test taken closest to A1c count used | Total hemoglobin concentration | -- | {OLIS} | -- |
| *Stratified groups* | At the estimated date of conception | Maternal age greater than or equal to 40 years | -- | {RPDB} | -- |
|  | At any point prior to the estimated date of conception | Diabetes mellitus | -- | {Ontario Diabetes Database (ODD) identifies women who have been diagnosed with diabetes mellitus} | <https://www.ncbi.nlm.nih.gov/pubmed/11874939> |
|  | At the estimated date of conception | Chronic hypertension | 401, 405, 642.0-642.2, 642.7 [I10, I15, O10, O11] | 401 | <https://www.ncbi.nlm.nih.gov/pubmed/19858407> |
|  | Same | Urban or rural residence | -- | {Statistics Canada census data} | -- |
|  | Same | Residential income quintile | -- | {Statistics Canada census data} | -- |
|  | Same | Multifetal pregnancy | M_MULTIBIRTH=’T’ or B_MULTIBIRTH=’T’ in MOMBABY | -- | -- |
|  | Same | Nulliparity or parity | M_PREVBIRTH_DERIVED in MOMBABY | -- | -- |
|  | End of the index pregnancy | Multifetal pregnancy | M_MULTIBIRTH=’T’ or B_MULTIBIRTH=’T’ in MOMBABY | -- | -- |
|  | Same | Nulliparity or parity | PREVTERM+PREVPRETERM | -- | -- |
|  | Same | Livebirth or stillbirth | M_STILLBIRTH=’F’ in MOMBABY (see <https://datadictionary.ices.on.ca/Applications/DataDictionary/Library.aspx?Library=MOMBABY>) | -- | -- |
|  | Same | Gestational diabetes mellitus | O24 | -- | -- |

^a^ Available for deliveries between April 2006 and March 2014.

BIS: BORN Information System; BORN: Better Outcomes Registry & Network; CCI: Canadian Classification of Interventions; DAD: Discharge Abstract Database; ICD-9: International Classification of Diseases, 9th Revision; ICD-10-CA: International Classification of Diseases, 10th Revision, Canada; IRCC: Immigration, Refugees and Citizenship Canada; ODD: Ontario Diabetes Dataset; OHIP: Ontario Health Insurance Plan; OLIS: Ontario Laboratories Information System
