# Supplementary material for: Risk of severe maternal morbidity or death in relation to elevated hemoglobin A1c preconception, and in early pregnancy: A population-based cohort study
Source: PLoS Med. 2020 May 19;17(5):e1003104. doi: 10.1371/journal.pmed.1003104 (PMC7236974; doi:10.1371/journal.pmed.1003104)
Supplement: S2 Table — All data shown are as a number (%) unless otherwise noted. A1c, hemoglobin A1c (DOCX) [file pmed.1003104.s006.docx]

**S2 Table.** **Characteristics of the preconception A1c sub-cohort and the non-A1c cohort, with standardized differences (*additional analysis 10*).** All data shown are as a number (%) unless otherwise noted.

| **Characteristic** | **Preconception sub-cohort (N = 31,225)** | **Non-A1c cohort**  **(N = 1,089,711)** | **Standardized difference** |
| --- | --- | --- | --- |
| **Maternal characteristic at the time of A1c testing** |  |  |  |
| Mean (SD) age, years | 31.1 (5.1) | 29.5 (5.4) | 0.31 |
| Median (IQR) parity | 1.0 (0.0-1.0) | 1.0 (0.0-1.0) | 0.01 |
| Nulliparous | 13,527 (43.3) | 475,970 (43.7) | 0.01 |
| Maternal world region of origin |  |  |  |
| Canada / long-term resident / unknown | 19,307 (61.8) | 793,389 (72.8) | 0.24 |
| Caribbean | 610 (2.0) | 15,668 (1.4) | 0.04 |
| East Asia / Pacific | 2389 (7.7) | 71,703 (6.6) | 0.04 |
| Hispanic America | 918 (2.9) | 22,724 (2.1) | 0.05 |
| Middle East / North Africa | 1464 (4.7) | 31,621 (2.9) | 0.09 |
| South Asia | 4187 (13.4) | 85,969 (7.9) | 0.18 |
| Sub-Saharan Africa | 866 (2.8) | 21,879 (2.0) | 0.05 |
| Western Nations / Europe | 1484 (4.8) | 46,758 (4.3) | 0.02 |
| Residing in the lowest income quintile area | 7119 (22.8) | 244,625 (22.4) | 0.01 |
| Rural or unknown residence | 1721 (5.5) | 112,783 (10.3) | 0.18 |
|  |  |  |  |
| **Maternal conditions at any point prior to conception** |  |  |  |
| Diagnosed diabetes mellitus | 3150 (10.1) | 11,488 (1.1) | 0.40 |
|  |  |  |  |
| **Maternal conditions within 1 year prior to conception** |  |  |  |
| Illegal drug or tobacco use | 896 (2.9) | 52,800 (4.8) | 0.10 |
| Chronic hypertension | 3798 (12.2) | 96,404 (8.8) | 0.11 |
| Mean (SD) body mass index, kg/m^2,a^ | 27.0 (7.1) | 25.7 (9.2) | 0.16 |
|  |  |  |  |
| **Characteristics of the index delivery** |  |  |  |
| Multibirth | 633 (2.0) | 21,014 (1.9) | 0.01 |
|  |  |  |  |
| **From 23 weeks’ gestation up to 42 days postpartum** |  |  |  |
| Total with SMM or death | 682 (2.2) | 17,489 (1.6) | 0.04 |

^a^ Among 7203 pregnancies in the preconception A1c sub-cohort and 285,898 pregnancies in the non-A1c cohort, with a recorded pre-pregnancy body mass index.
