# Supplementary material for: Risk of severe maternal morbidity or death in relation to elevated hemoglobin A1c preconception, and in early pregnancy: A population-based cohort study
Source: PLoS Med. 2020 May 19;17(5):e1003104. doi: 10.1371/journal.pmed.1003104 (PMC7236974; doi:10.1371/journal.pmed.1003104)
Supplement: S3 Table — SMM indicators are separated into those with a likely, possibly, or unlikely relation to maternal average glucose concentration. Specific ICD-10-CA or CCI codes are identified, and where necessary, references are provided. CCI, Canadian Classification of Interventions; ICD-10-CA, International Classification of Diseases, 10th Revision, Canada; SMM, severe maternal morbidity. (DOCX) [file pmed.1003104.s007.docx]

**S3 Table.** **Further information on the plausible relation between pre-pregnancy maternal glycemia and the various indicators of severe maternal morbidity (SMM)^1^ (for *additional analysis 11)*.** SMM indicators are separated into those with a **likely**, **possibly**, or **unlikely** relation to maternal average glucose concentration. Specific ICD-10-CA or CCI codes are identified, and where necessary, references are provided.

| **Indicator Group** | **SMM Indicator** | **ICD-10-CA or CCI Codes** | **Reference** |
| --- | --- | --- | --- |
| *SPE, HELLP, Eclampsia* | Severe pre-eclampsia, HELLP syndrome | O14.1, or O14.2 | 2 |
|  | Eclampsia | O15 | 2 |
| *Severe Hemorrhage* | Placenta previa with hemorrhage and red cell transfusion | O44.1 + CIHI BTREDBC = 1 |  |
|  | Placental abruption with coagulation defect | O45.0 |  |
|  | Antepartum hemorrhage with coagulation defect | O46.0 | 15 |
|  | Intrapartum hemorrhage with coagulation defect | O67.0 | 15 |
|  | Intrapartum hemorrhage with red cell transfusion | O67 + CIHI BTREDBC = 1 | 15 |
|  | Postpartum hemorrhage with red cell transfusion, procedures to the uterus or hysterectomy | O72 + any of the following: BTREDBC = 1, or (1.RM.13, 1.KT.51, 5.PC.91.LA or 5.PC.91.HV) + BTREDBC = 1, or (5.MD.60.RC, 5.MD.60.RD, 5.MD.60.KE, 5.MD.60.CB or 1.RM.89.LA), or 1.RM.87.LA-GX. **NOTE**: **1.RM.89.LA** is included only if codes 1.PL.74, 1.RS.74 or 1.RS.80 are NOT also present. | 15 |
|  | Curettage with red cell transfusion | (5.PC.91.GA, 5.PC.91.GC, 5.PC.91.GD) + CIHI BTREDBC = 1 |  |
| *Maternal ICU Admission* | Maternal ICU admission | SCU in (‘10’, ’20’, ’25’, ’30’, ’35’, ’40’,’45’,’60’,’80’) | 3 |
| *Surgical Complications* | Complications of obstetric surgery and procedures | O75.4 | 12 |
|  | Evacuation of incisional hematoma with RBC transfusion | 5.PC.73.JS + CIHI BTREDBC = 1 |  |
|  | Repair of bladder, urethra, or intestine | 5.PC.80.JR, 1.NK.80, 1.NM.80 |  |
|  | Reclosure of caesarean wound with RBC transfusion | (5.PC.80.JM, 5.PC.80.JH) + CIHI BTREDBC = 1 |  |
|  | Procedures to the uterus with RBC transfusion | (1.RM.13, 1.KT.51, 5.PC.91.LA, 5.PC.91.HV) + CIHI BTREDBC = 1 |  |
| *Hysterectomy* | Caesarean hysterectomy, hysterectomy using an open approach | 5.MD.60.RC, 5.MD.60.RD, 5.MD.60.KE, 5.MD.60.CB, 1.RM.89.LA (exclude if 1.PL.74, 1.RS.74 or 1.RS.80 code also present), 1.RM.87.LA-GX |  |
| *Sepsis* | Puerperal sepsis | O85 | 4, 5 |
|  | Septicemia during labour | O75.3 | 4, 5 |
| *Embolism, Shock, DIC* | Obstetric embolism | O88 | 14 |
|  | Obstetric shock | O75.1, R57, T80.5 or T88.6 |  |
|  | Disseminated intravascular coagulation | D65 | 7 |
| *Assisted Ventilation* | Assisted ventilation through endotracheal tube | 1.GZ.31.CA-ND |  |
|  | Assisted ventilation through tracheostomy | 1.GZ.31.CR-ND |  |
| *Cardiac Conditions* | Cardiomyopathy, cardiac arrest and resuscitation, myocardial infarction, pulmonary edema and heart failure | O74.2, O89.1, O90.3, I21, I22, I42, I43, I46, I49.0, I50, J81, 1.HZ.09 or 1.HZ.30 | 8, 9 |
| *Acute Renal Failure* | Acute renal failure | O90.4, N17, N19 or N99.0 | 6 |
|  | Dialysis | 1.PZ.21 | 6 |
| *Severe Uterine Rupture* | Rupture of the uterus with red cell transfusion, procedures to the uterus or hysterectomy | (O71.0 or O71.1) + any of the following: CIHI BTREDBC = 1, or (1.RM.13, 1.KT.51, 5.PC.91.LA or 5.PC.91.HV) + CIHI BTREDBC = 1, or (5.MD.60.RC, 5.MD.60.RD, 5.MD.60.KE, 5.MD.60.CB or 1.RM.89.LA**^a^**), or 1.RM.87.LA-GX. **NOTE**: 1.RM.89.LA is included only if codes 1.PL.74, 1.RS.74 or 1.RS.80 are NOT also present | 13 |
| *Cerebrovascular Accidents* | Cerebral venous thrombosis in pregnancy | O22.5 | 14 |
|  | Cerebral venous thrombosis in the puerperium | O87.3 | 14 |
|  | Subarachnoid and intracranial hemorrhage, cerebral infarction | I60, I61, I62, I63, or I64 | 17 |
|  | Acute fatty liver with red cell transfusion or plasma transfusion | O26.6 + (CIHI BTREDBC = 1 or CIHI BTPLASMA = 1) | 16 |
|  | Cerebral edema or coma | G93.6 or R40.2 | 19 |
| *Miscellaneous* | Pulmonary, cardiac, and CNS complications of anesthesia during pregnancy, labour, delivery or the puerperium | O29.0, O29.1, O29.2, O89.0, O89.1, O89.2, O74.0, O74.1, O74.2 or O74.3 | 12 |
|  | Status asthmaticus | J45.01, J45.11, J45.81 or J45.91 | 10 |
|  | Adult respiratory distress syndrome | J80 | 3 |
|  | Hepatic failure | K71 or K72 | 18 |
|  | Acute abdomen | K35, K37, K65, N73.3 or N73.5 |  |
|  | Surgical or manual correction of inverted uterus for vaginal births only | 5.PC.91.HQ or 5.PC.91.HP, restricted to vaginal births (i.e., absence of caesarean 5.MD.60) |  |
|  | Sickle-cell anemia with crisis | D57.0 |  |
|  | Acute psychosis | F53.1 or F23 |  |
|  | Status epilepticus | G41 |  |

*References for S3 Table*

1. Dzakpasu S, Deb-Rinker P, Arbour L, Darling EK, Kramer MS, Liu S, et al. Severe Maternal Morbidity in Canada: Temporal Trends and Regional Variations, 2003-2016. J Obstet Gynaecol Can 2019;41: 1589-1598.e16. https://doi.org/10.1016/j.jogc.2019.02.014

2. Cavero-Redondo I, Martinez-Vizcaino V, Soriano-Cano A, et al. Glycated haemoglobin A1c as a predictor of preeclampsia in type 1 diabetic pregnant women: A systematic review and meta-analysis. Pregnancy Hypertens 2018; 14:49-74. https://doi.org/10.1016/j.preghy.2018.04.004.

3. Inkster ME, Fahey TP, Donnan PT, et al. Poor glycated haemoglobin control and adverse pregnancy outcomes in type 1 and type 2 diabetes mellitus: Systematic review of observational studies. BMC Pregnancy Childb 2006; 6:30-43. doi:10.1186/1471-2393-6-30

4. Axelsson D, Blomberg M. Maternal obesity, obstetric interventions and post-partum anaemia increase the risk of post-partum sepsis: a population-based cohort study based on Swedish medical health registers. Infect Dis 2017; 49:765-771. doi: 10.1080/23744235.2017.1341055

5. Acosta CD, Bhattacharya S et al. Maternal sepsis: a Scottish population-based case–control study. BJOG 2012; 119:474-483. doi: 10.1111/j.1471-0528.2011.03239.x

6. Girman CJ, Kou TD, Brodovicz K, et al. Risk of acute renal failure in patients with type 2 diabetes mellitus. Diabet Med 2012; 29:614-21. doi: 10.1111/j.1464-5491.2011.03498.x.

7. Nogami K, Muraki I, Imano H, Iso H. Risk of disseminated intravascular coagulation in patients with type 2 diabetes mellitus. BMJ Open 2017; 7:e013894. doi: 10.1136/bmjopen-2016-013894.

8. Boudina S, Abel ED. Diabetic cardiomyopathy, causes and effects. Rev Endocr Metab Disord 2010; 11:31-9. https://doi.org/10.1007/s11154-010-9131-7

9. Poirier P, Giles TD, Bray GA, et al. Obesity and Cardiovascular Disease: Pathophysiology, Evaluation, and Effect of Weight Loss. Circulation 2006; 113:898-918. https://doi.org/10.1161/CIRCULATIONAHA.106.171016.

10. Monahan S, Tapp H, McWilliams A, Dulin M. Obesity and asthma: Pathophysiology and implications for diagnosis and management in primary care. Exp Biol Med 2014; 239:1531-40. doi: 10.1177/1535370214525302

11. Dagogo-Jack S, Alberti KGMM. Management of Diabetes Mellitus in Surgical Patients. Diabet Spec 2002; 15:44-48. https://doi.org/10.2337/diaspect.15.1.44.

12. McAnulty GR, Robertshaw HJ, Hall GM. Anaesthetic management of patients with diabetes mellitus. Brit J Anaesth 2000; 85:80-90. https://doi.org/10.1093/bja/85.1.80

13. Ofir K, Sheiner E, Levy A, et al. 2003. Uterine rupture: Risk factors and pregnancy outcome. AJOG 2003; 189:1042-1046. https://doi.org/10.1067/S0002-9378(03)01052-4.

14. Jacobsen AF, Skjeldestad FE, Sandset PM. Ante‐ and post-natal risk factors of venous thrombosis: a hospital‐based case–control study. J Thromb Haemost 2008; 6:905-912. https://www.ncbi.nlm.nih.gov/pubmed/18363820

15. Sebghatti M, Chandraharan E. An update on the risk factors for and management of obstetric haemorrhage. Womens Health 2017; 13:34-40. doi: 10.1177/1745505717716860.

16. Bhatt HB, Smith RJ. Fatty liver disease and diabetes mellitus. Hepatobillary Surg Nutr 2015; 4:101-108 https://dx.doi.org/10.3978%2Fj.issn.2304-3881.2015.01.03

17. Pezzini A, Grassi M, Paciaroni M, et al. Obesity and the Risk of Intracerebral Hemorrhage. Stroke 2013; 44:1584-1589. https://doi.org/10.1161/STROKEAHA.111.000069

18. Tolman KG, Fonseca V, Dalpiaz A, Tan MH. Spectrum of Liver Disease in Type 2 Diabetes and Management of Patients with Diabetes and Liver Disease. Diabetes Care 2007; 30:734-743. https://doi.org/10.2337/dc06-1539

19. Siwakoti K, Giri S, Kadaria D. Cerebral edema among adults with diabetic ketoacidosis and hyperglycemic hyperosmolar syndrome: Incidence, characteristics, and outcomes. J Diabetes 2017; 9:208-209. https://doi.org/10.1111/1753-0407.12448
