# Supplementary material for: Risk of severe maternal morbidity or death in relation to elevated hemoglobin A1c preconception, and in early pregnancy: A population-based cohort study
Source: PLoS Med. 2020 May 19;17(5):e1003104. doi: 10.1371/journal.pmed.1003104 (PMC7236974; doi:10.1371/journal.pmed.1003104)
Supplement: S4 Table — Green indicates a strong theoretical relation, yellow indicates a possible relation, and red indicates an unlikely relation, as outlined in S1 Table. RRs were adjusted for maternal age and world region of origin—each at the time of the A1c test—as well as drug or tobacco dependence <1 year before conception and multifetal pregnancy. This analysis comprises 31,225 pregnancies in the preconception sub-cohort. A1c, hemoglobin A1c; RR, relative risk; SMM, severe maternal morbidity (DOCX) [file pmed.1003104.s008.docx]

**S4 Table. Risk of severe maternal morbidity (SMM) between 23 weeks’ gestation up to 42 days postpartum likely, possibly and unlikely to be related to a 0.5% absolute increase in preconception A1c (*additional analysis 11*).** Green indicates a strong theoretical relation, yellow indicates a possible relation, and red indicates an unlikely relation, as outlined in **S1 Table**. Relative risks were adjusted for maternal age and world region of origin -- each at the time of the A1c test -- as well as drug or tobacco dependence < 1 year before conception and multifetal pregnancy. This analysis comprises 31,225 pregnancies in the preconception sub-cohort.

| **Relation of SMM bundle to maternal average glucose concentration** | **No. (overall %) with SMM or death** | **Unadjusted relative risk (95% CI)** | ***p*-Value** | **Adjusted relative risk (95% CI)** | ***p*-Value** |
| --- | --- | --- | --- | --- | --- |
| *Likely* | 331 (1.1) | 1.17 (1.14 to 1.21) | < 0.001 | 1.17 (1.13 to 1.20) | < 0.001 |
| *Possibly* | 257 (0.82) | 1.19 (1.15 to 1.23) | < 0.001 | 1.19 (1.15 to 1.23) | < 0.001 |
| *Unlikely* | 93 (0.30) | 0.97 (0.85 to 1.12) | 0.72 | 0.93 (0.78 to 1.11) | 0.42 |
