# Supplementary material for: Risk of severe maternal morbidity or death in relation to elevated hemoglobin A1c preconception, and in early pregnancy: A population-based cohort study
Source: PLoS Med. 2020 May 19;17(5):e1003104. doi: 10.1371/journal.pmed.1003104 (PMC7236974; doi:10.1371/journal.pmed.1003104)
Supplement: S1 Prospective Protocol — ICES, Institute for Clinical and Evaluative Sciences. (DOCX) [file pmed.1003104.s009.docx]

| Project InitiationThis Section must be Completed Prior to Project Dataset(s) Creation | | | | | |
| --- | --- | --- | --- | --- | --- |
| **Project Title:** | Pre-Pregnancy Renal Function and Outcomes | | | | |
| **Project TRIM number:** | 2019 0900 169 000 | | | | |
| **Research Program:** | HSPE | | | | |
| **Site:** | ICES Central | | | | |
| **Project Objectives:** | *Insert Project Objectives as listed in the approved ICES Project PIA* | | | | |
|  | **Objective 4:** To evaluate the association between pre-pregnancy glycemic control (hemoglobin A1c [A1c]) and severe maternal morbidity (SMM) or death. | | | | |
| **ICES Project PIA Initial Approval Date:** | *The ICES Employee or agent who is responsible for creating the Project Dataset(s) is responsible for ensuring there is an approved ICES Project PIA and verifying the date of approval prior to creating the Project Dataset(s)* | | | | |
|  | 2018-10-12 | | | | |
| **Principal Investigator (PI):** | Joel Ray, Ziv Harel | | | | |
| **Check the applicable box if the PI is an ICES Student/Trainee** | ICES Student  ICES Fellow  ICES Post-Doctoral Trainee  Visiting Scholar | | | | |
| **Responsible ICES Scientist:** | *Name the Responsible ICES Scientist if the PI is not a Full Status ICES Scientist* | | | | |
|  | Joel Ray | | | | |
| **Project Team Member(s) Responsible for Project Dataset Creation and/or Statistical Analysis and date joined (list all):** | *All person(s) (ICES Analyst, Appointed Analyst, Analytic Epidemiologist, PI, and/or Student) responsible for creating the Project Dataset(s) and/or statistical analysis on the Research Analytics Environment (RAE) and the date they joined the project must be recorded* | | | | |
|  | Ziv Harel  Joel Ray  Alison Park  Alexander Davidson (summer student with Joel) | | | 2018-Oct  2018-Oct  2018-Oct  2019-Jun | |
| **Other ICES Project Team Members and date joined (list all):** | *All other Research Project Team Members (e.g., Research Administrative Assistants, Research Assistants, Project Managers, Epidemiologists) and the date they joined the project must be recorded* | | | | |
|  |  | | | yyyy-mon-dd | |
| **Confirmation that DCP is consistent with Project Objectives:** | *The following individuals must confirm that the ICES Data provided for in this DCP is relevant (e.g., with respect to cohort, timeframe, and variables) and required to achieve the Project Objectives stated in the ICES Project PIA prior to initial Project Dataset creation: 1) PI; 2) Responsible ICES Scientist if the PI is not a Full Status ICES Scientist, or a second ICES Scientist or the Scientific Program Lead if the PI is creating both the DCP and the Project Dataset[s]; 3) ICES Research and Analysis Staff creating the DCP; and 4) ICES Analytic Staff (ICES Employee or agent responsible for creating the Project Dataset[s]). This may be delegated either verbally or via e-mail.* | | | | |
|  | ***Principal Investigator***  Joel Ray | |  | |  |
|  | ***Responsible ICES Scientist or Second ICES Scientist/Lead*** | |  |  | |
|  | ***ICES Research and Analysis Staff Creating the DCP***  Alison Park | |  | 2019-Jun-17 | |
|  | ***ICES Analytic Staff***  Alison Park | |  | 2019-Jun-17 | |
| **Designated ICES Research and Analysis Staff accountable for Project Documentation:** | *The person named (ICES staff) is accountable for ensuring that the approved ICES Project PIA, ICES Project PIA Amendments, and DCP are saved on the T Drive, ensuring ICES Project PIA Amendments are submitted as required, ensuring DCP Amendments are documented, and sharing the final DCP with the PI/Responsible ICES Scientist at project completion* | | | | |
|  | Alison Park | | | | |
| **DCP Creation Date and Author:** | *Date DCP was finalized prior to Project Dataset(s) creation* | *Name of person who created the DCP* | | | |
|  | ***Date*** | ***Name*** | | | |
|  | 2019-jun-14 | Objective #4: Alison Park | | | |

| ICES DataThis Section must be Completed Prior to Project Dataset(s) Creation | |
| --- | --- |
| *The ICES Employee or agent who is responsible for creating the Project Dataset(s) must ensure that this list includes only data listed in the ICES Project PIA*  *Changes to this list after initial ICES Project PIA approval require an ICES Project PIA Amendment* | *Mandatory for all datasets that are available by individual year* |
| ***General Use Datasets – Health Services*** | ***Years (where applicable)*** |
| OHIP | 2002-2016 |
| CIHI DAD | 2002-2016 |
| CIHI SDS | 2002-2016 |
| NACRS | 2002-2016 |
| ***General Use Datasets – Population*** |  |
| RPDB | 2002-2016 |
| ***General Use Datasets – Coding/Geography*** |  |
| REF | - March 2011 |
| PCCF | 2002-2009 |
| ***General Use Datasets - Other*** |  |
| MOMBABY | 2002-2017 |
| ODD | 2002-2016 |
| CORR | 2002-2014 |
| ***Other Datasets*** |  |
| CIC | 2006-2016 |
| BORN-Niday and BIS | 2006/07-2013/14 |
| OLIS | March 2007-September 2015 |

| Project Amendments and Reconciliation | | | |  |  |
| --- | --- | --- | --- | --- | --- |
| **ICES Project PIA Amendment History (add additional rows as needed):** | *Privacy approval date* | *Person who submitted amendment* | *Note that any changes to the list of ICES Data or Project Objectives require an ICES Project PIA Amendment* |  |  |
|  | ***Date*** | ***Name*** | ***Amendment*** |  |  |
|  | 2019-06-XX | Alison | Objective 4 -- pending |  |  |
|  |  |  |  |  |  |
| **DCP Amendment History (add additional rows as needed):** | *Date DCP amended* | *Person who made the DCP amendment* | *Note that any DCP amendments involving changes to the list of ICES Data or Project Objectives require an ICES Project PIA Amendment* |  |  |
|  | ***Date*** | ***Name*** | ***Amendment*** |  |  |
|  | 2019-06-14 | Alison | Objective 4 |  |  |
| **Date Programs/DCP reconciled** | *The person(s) creating the dataset and/or analyzing the data are responsible for ensuring that the final DCP reflects the final program(s) when the project is completed* | | |  |  |
|  | yyyy-mon-dd | | |  |  |
|  |  | | |  |  |

| Project Cohort | | |
| --- | --- | --- |
| **Study Design** | Cohort study  Matched cohort study  Case-control study  Cross-sectional study  Other (specify): | |
| **Index Event / Inclusion Criteria** | Women with any obstetric delivery in Ontario from March 2007 onward, and who underwent HbA1c screening in the first 90 days before conception, or within 19 weeks following conception (i.e. up to 21 completed weeks’ gestation), between March 2007 and Dec 2015, will form the **screened cohort**. Those who did not undergo A1c screening during the same period will form the **non-screened cohort**.  The estimated date of conception should be calculated as [B_BDATE/M_ADMDATE] minus the B_GESTWKS_DEL [else M_GESTWKS_DEL]*7 in MOMBABY + 14 days.  For each mom having multiple pregnancies the analyses will account for the nesting of >1 potential pregnancy in a given woman. | |
| **Estimated Size of Cohort**  **(if known)** | In a feasibility analysis, there are 105,737 pregnancies with an outpatient HbA1c test at -10 to +20 weeks gestation:   - 24693 pregnancies have A1c before conception - 87071 pregnancies have A1c within pregnancy - 6027 pregnancies have A1c both before and after conception | |
| **Exclusions (in order)** | *Step* | Description |
|  | 1 | Invalid OHIP number or otherwise ineligible for OHIP   1. Invalid or missing M_IKN, M_KEY, B_IKN or B_KEY (i.e., WARN^=’N’) . 2. Missing age or sex for mother in RPDB 3. Maternal sex = male in RPDB |
|  | 2 | Non-Ontario resident at the time of the index conception |
|  | 2 | Mother’s age < 16 years or > 50 years at the time of the index conception (getdemo) |
|  | 3 | Mother’s death prior to 23 completed weeks’ gestation. |
|  | 4 | Missing [M_GESTWKS_DEL] and [B_GESTWKS_DEL] in MOMBABY at the time of the index delivery |
|  | 5 | Delivery before 23 completed weeks’ gestation |

| Project Time Frame Definitions | | |
| --- | --- | --- |
| Look-back Window  Observation Window  (in which to look for outcomes)  **Index Event Date**  Accrual Window  Max Follow-up Date | |  |
| **Accrual Start/End Dates** | MOMBABY Apr 2007-Oct 2016;  OLIS Mar 2007-Sep 2015 |  |
| **Max Follow-up Date** | Dec 31, 2016 |  |
| **When does observation window terminate?** | Earliest of:  1. 42 days after the index delivery date [B_BDATE/M_ADMDATE]  2. Max f/u date (Dec 31, 2016)  3. Any of the outcomes (below) |  |
| **Lookback Window(s)** | One year preceding the estimated date of conception, lookback for tobacco use, diabetes and chronic hypertension |  |

| Variable Definitions (add additional rows as needed) | | |
| --- | --- | --- |
| **Main Exposure or Risk Factor** | Outpatient hemoglobin A1c in OLIS* expressed as a percent of total hemoglobin and analyzed in 0.5% increments:    observationcode = "17855-8”, “17856-6”, “41995-2”, “4548-4”, “71875-9” expressed as percentage of total hemoglobin. Restrict to values between 2-20%.  observationcode = “59261-8” is expressed as mmol/mol. Convert to % using formula: *HbA1c( as a %) = [HbA1c(in mmol/mol)/10.929] + 2.15*  *Outpatient in OLIS defined as the value not occuring during a hospital admission, emergency room visit (via NACRS) or discharge date.  Two exposure periods:   1. **Preconception**: At least one outpatient HbA1c value in OLIS in the first 90 days before conception. 2. **Within pregnancy**: At least one outpatient HbA1c value in OLIS from 0 to 19^6/7^ weeks’ after conception.   NOTE: Where a given woman had more than one A1c test, the one nearest to conception was used in the **preconception** analysis. The latest A1c test was used **within pregnancy**. |  |
| **Primary Outcome Definition** | Severe Maternal Morbidity (SMM) or death arising from 23^0/7^ weeks’ gestation up to 42 days after the index birth. |  |
| **Secondary Outcome Definition(s)** | SMM or death arising from the index birth up to 42 days thereafter |  |
| **Baseline Characteristics** | See Table 1 |  |
| **Other** |  |  |

| Analysis Plan and Dummy Tables |
| --- |
| **Main model:**  Plot the continuous relation between preconception A1c, in 0.5% increments, and the probability of SMM or death from 23^0/7^ weeks’ gestation up to 42 days after birth. Covariates are maternal age, multifetal pregnancy, world region of origin—all at the time of A1c screening—as well as tobacco or illegal drug dependence within one year preceding the estimated date of conception.  **Additional analysis 1:**  Restrict the main model to a subset of women with a recorded BMI at the time of their preconception A1c screening, and add as a covariate to the main model.  **Additional analysis 2:**  As there is a potential effect of maternal anemia on A1c concentration, the earliest maternal hemoglobin concentration in pregnancy was also added as a covariate to the main model.  **Additional analysis 3:**  Stratify the main model by:   - nulliparous vs parous - singleton vs multifetal - livebirth vs stillbirth - age ≥ 40 years vs < 40 years (at conception) - residential income quintile (at conception) - urban vs rural residence (at conception) - pre-pregnancy diabetes mellitus (identified up to 1 year before conception) - chronic hypertension (up to 1 year before conception)   **Additional analysis 4:**  Re-run main model analysing **preconception** A1c and subsequent SMM or death occurring only from birth up to 42 days  **Additional analysis 5:**  Re-run main model using A1c measured **within pregnancy,** between the date of conception and up to 19 completed weeks’ gestation. If a woman had more than one A1c measured in this time period, then the latest value was chosen, to minimize the temporal separation of average glycemic control and the potential onset of SMM.  **Supplementary Table 2:**  A woman who undergoes A1c screening may differ from one that does not. To address this, select women in the **non-screened cohort** who otherwise met the same criteria as those in the **preconception** exposure group. Compare baseline variables between the **non-screened** and **preconception** cohorts using standardized differences.  **Additional analysis 6:**  Some SMM indicators may be more plausibly related to maternal glycemic control than others. Accordingly, two authors (AD and JR) informally searched the literature and classified the SMM indicators into three groups: those likely, possibly, or unlikely to be related to A1c (**Supplementary Table 3**). Thus, the main model of **preconception** A1c was compared with the **likely**, **possible** or **unlikely** outcome of SMM. Run a multinomial logistic regression for the association between preconception A1c and 1) SMM likely related to A1c, 2) SMM possibly related to A1c, 3) SMM unlikely to be related to A1c, relative to 4) no SMM. Where a woman has > 1 SMM, use the following hierarchy to classify her SMM as Likely/Possibly/Unlikely to be related to A1c: If the woman had any SMM that was likely related to A1c then classify her outcome as “Likely”, else if she had any SMM considered possibly related to A1c, then classify her outcome as “Possible”, else if she had any SMM considered unlikely related to A1c then classify her outcome as “Unlikely”. |

**Table 1.** Characteristics of pregnant women in the screened cohort who underwent A1c testing from minus 90 days up to conception (**preconception**) and women tested from conception up to 19 weeks thereafter (**within pregnancy**). All data are shown as a number (%) unless otherwise noted.

| **Characteristic** | **Preconception**  **(N = )** | **Within pregnancy**  **(N = )** | **Standardized difference** |
| --- | --- | --- | --- |
| *From 23 weeks’ gestation up to 42 days after the index delivery* |  |  |  |
| Total with SMM or death |  |  |  |
| Median number of SMM indicators (IQR) |  |  |  |
| *At the time of A1c screening* |  |  |  |
| Mean maternal age, years |  |  |  |
| Maternal world region of origin |  |  |  |
| First |  |  |  |
| Second |  |  |  |
| Third |  |  |  |
| Fourth |  |  |  |
| Other |  |  |  |
| Multifetal pregnancy |  |  |  |
| Parity, median (IQR) |  |  |  |
| Nulliparity |  |  |  |
| Rural residence |  |  |  |
| Residence in the lowest income quintile area |  |  |  |
| Mean maternal A1c, % |  |  |  |
| Women < 5.8% A1c |  |  |  |
| Women 5.8%-6.4% A1c |  |  |  |
| Women > 6.4% A1c |  |  |  |
| Mean maternal pre-pregnancy BMI^a^, kg/m^2^ |  |  |  |
| Diabetes mellitus |  |  |  |
| *Conditions in year before index delivery* |  |  |  |
| Illegal drug or tobacco use |  |  |  |
| Chronic hypertension |  |  |  |

^a^ Limited to XX,XXX women for whom there was a recorded pre-pregnancy height and weight

**Figure 2** - Plot of continuous relation between probability of SMM or death (y-axis) with increasing 0.5% increments of A1c (x-axis). Might combine histogram of A1c with this graph.

**Figure 3** - Forest plot of the relative risk of SMM or death per 0.5% increment of **preconception** A1c, stratified into several categories

**Figure 4** - Forest plot of the relative risk of SMM or death per 0.5% increment of **within pregnancy** A1c, stratified into the same categories

**Figure 5** - Forest plot of SMM as colour-coded

| **Supplementary Table 1.** Variables used to define cohort entry and exclusion criteria, as well as study exposures, outcomes, adjustment, and stratification. | | | | | |
| --- | --- | --- | --- | --- | --- |
| **Assessment** | **Timing** | **Disease, procedure or condition** | **ICD-10-CA or CCI codes** | **OHIP ICD-9 diagnostic codes or fee codes {or other source if in parentheses}** | **PubMed link to related validation studies for some codes** |
| *Cohort entry criteria* | Jan 2007-Sep 2015 | Pregnant women who underwent A1c screening | -- | {OLIS} | -- |
| *Exclusion criteria* | At the estimated time of conception | Maternal age greater than 50 years or less than 16 years | -- | {RPDB} | -- |
|  | At the time of A1c screening | Non-Ontario resident, invalid OHIP number, otherwise ineligible for OHIP | -- | {RPDB} | -- |
|  | Prior to 23 weeks’ gestation | Mother passed away | -- | {RPDB} | -- |
| *Study exposure* | Up to 90 days before the estimated date of conception (14 days after LMP). Select latest test if > 1. | Preconception hemoglobin A1c | -- | {OLIS} | -- |
|  | From the estimated date of conception (14 days after LMP) up to 19 weeks after (i.e. 21 weeks’ gestation). Select latest test if > 1. | Within pregnancy hemoglobin A1c | -- | {OLIS} | -- |
| *Study outcome* | From 23 weeks prior to the index birth to 42 days following birth | Severe maternal morbidity (SMM) or death | Severe preeclampsia and HELLP syndrome: O14.1 or O14.2  Eclampsia: O15  Cerebral venous thrombosis in pregnancy, or in the puerperium: O22.5, or O87.3  Acute fatty liver with red blood cell (RBC) transfusion or plasma transfusion: O26.6  + (CIHI BTREDBC = 1 or CIHI BTPLASMA = 1)  Pulmonary, cardiac, and CNS complications of anaesthesia during pregnancy, the puerperium, or labour and delivery: O29.0,  O29.1, O29.2, O89.0, O89.1, O89.2, O74.0, O74.1, O74.2 or O74.3  Placenta previa with hemorrhage with RBC transfusion: O44.1 + CIHI BTREDBC = 1  Placental abruption with coagulation defect: O45.0  Antepartum hemorrhage with coagulation defect: O46.0    Intrapartum hemorrhage with coagulation defect: O67.0  Intrapartum hemorrhage with RBC transfusion: O67 + CIHI BTREDBC = 1  Rupture of the uterus with RBC transfusion, procedures to the uterus or hysterectomy:  (O71.0 or O71.1) + any of the following:   - CIHI BTREDBC = 1, or - (1.RM.13, 1.KT.51, 5.PC.91.LA or 5.PC.91.HV) + CIHI BTREDBC = 1, or - (5.MD.60.RC, 5.MD.60.RD, 5.MD.60.KE, 5.MD.60.CB or 1.RM.89.LA**^a^**), or - 1.RM.87.LA-GX   **^a^ NOTE**: **1.RM.89.LA** is included only if codes 1.PL.74, 1.RS.74 or 1.RS.80 are NOT also present  Postpartum hemorrhage with RBC transfusion, procedures to the uterus or hysterectomy:  O72 + any of the following:   - BTREDBC = 1, or - (1.RM.13, 1.KT.51, 5.PC.91.LA or 5.PC.91.HV) + BTREDBC = 1, or - (5.MD.60.RC, 5.MD.60.RD, 5.MD.60.KE, 5.MD.60.CB or 1.RM.89.LA**^b^**), or - 1.RM.87.LA-GX   **^b^ NOTE**: **1.RM.89.LA** is included only if codes 1.PL.74, 1.RS.74 or 1.RS.80 are NOT also present  Cardiac conditions: O74.2, O89.1, O90.3, I21, I22, I42, I43, I46, I49.0, I50, J81, 1.HZ.09 or 1.HZ.30  Obstetric shock: O75.1, R57, T80.5 or T88.6  Septicemia during labour: O75.3  Complications of obstetric surgery and procedures: O75.4  Puerperal sepsis: O85  Obstetric embolism: O88  Acute renal failure: O90.4, N17, N19, N99.0  Death, obstetric, cause unspecified: O95  Death, obstetric, after 42 days but 1 year after delivery: O96  Death from sequelae of direct obstetric causes: O97    Disseminated intravascular coagulation: D65  Sickle cell anemia with crisis: D57.0  Acute psychosis: F53.1 or F23  Status epilepticus: G41  Cerebral edema or coma: G93.6 or R40.2  Cerebrovascular diseases: subarachnoid and intracranial hemorrhage, cerebral infarction, stroke: I60, I61, I62, I63 or I64  Status asthmaticus: J45.01, J45.11, J45.81 or J45.91  Adult respiratory distress syndrome: J80  Acute abdomen: K35, K37, K65, N73.3 or N73.5  Hepatic failure: K71 or K72  Sudden death, death from unspecified cause: R96, R97, R98 or R99  Assisted ventilation through endotracheal tube: 1.GZ.31.CA-ND  Assisted ventilation through tracheostomy:  1.GZ.31.CR-ND  Hysterectomy: 5.MD.60.RC, 5.MD.60.RD, 5.MD.60.KE, 5.MD.60.CB, 1.RM.89.LA (exclude if 1.PL.74, 1.RS.74 or 1.RS.80 code also present), 1.RM.87.LA-GX  Dialysis: 1.PZ.21  Evacuation of incisional hematoma with RBC transfusion: 5.PC.73.JS + CIHI BTREDBC = 1  Repair of bladder, urethra, or intestine: 5.PC.80.JR, 1.NK.80, 1.NM.80  Procedures to the uterus/pelvic vessels with RBC transfusion: (1.RM.13, 1.KT.51, 5.PC.91.LA, 5.PC.91.HV) + CIHI BTREDBC = 1  Surgical or manual correction of inverted uterus for vaginal births only: 5.PC.91.HQ or 5.PC.91.HP, restricted to vaginal births (i.e., absence of caesarean 5.MD.60)  Reclosure of caesarean wound with RBC transfusion: (5.PC.80.JM, 5.PC.80.JH) + CIHI BTREDBC = 1  Curettage with RBC transfusion: (5.PC.91.GA, 5.PC.91.GC, 5.PC.91.GD) + CIHI BTREDBC = 1  Maternal ICU admission: SCU in (‘10’, ’20’, ’25’, ’30’, ’35’, ’40’,’45’,’60’,’80’)  HIV disease: B24 | All cause death in {RPDB} | -- |
| *Covariates* | Within one year preceding the estimated date of conception | Tobacco or drug dependence | 291, 292, 2940, 303, 304, 305, 648.3, 649.0, 6555, 980 [F10-F19, F55, G312, O354, O355, T51, T652, Z720, Z721, Z722] | 291, 292, 303, 304, 305 | -- |
|  | At the estimated date of conception | Body mass index | -- | {BORN} | -- |
|  | Same | Maternal age | -- | {RPDB} | -- |
|  | Same | World region of origin | -- | {IRCC Permanent Resident Database } | -- |
|  | Same | Multifetal pregnancy | M_MULTIBIRTH=’T’ or B_MULTIBIRTH=’T’ in MOMBABY | -- |  |
|  | Test taken closest to A1c count used | Total hemoglobin concentration | -- | {OLIS} | -- |
| *Stratified groups* | At the estimated date of conception | Maternal age greater than or equal to 40 years | -- | {RPDB} | -- |
|  | Within 1 year preceding the estimated date of conception | Diabetes mellitus | 250, 648.8 [E10, E11, E13, E14, O244] | 250 or {ODD} | <https://www.ncbi.nlm.nih.gov/pubmed/11874939> |
|  | Within one year preceding the estimated date of conception | Chronic hypertension | 401, 405, 642.0-642.2, 642.7 [I10, I15, O10, O11] | 401 | <https://www.ncbi.nlm.nih.gov/pubmed/19858407> |
|  | Same | Urban or rural residence | -- | {Statistics Canada census data} | -- |
|  | Same | Residential income quintile | -- | {Statistics Canada census data} | -- |
|  | Same | Multifetal pregnancy | M_MULTIBIRTH=’T’ or B_MULTIBIRTH=’T’ in MOMBABY | -- | -- |
|  | Same | Nulliparity or parity | M_PREVBIRTH_DERIVED in MOMBABY | -- | -- |
|  | End of pregnancy | Livebirth or stillbirth | M_STILLBIRTH=’F’ in MOMBABY (see <https://datadictionary.ices.on.ca/Applications/DataDictionary/Library.aspx?Library=MOMBABY>) | -- | -- |

BORN: Better Outcomes Registry & Network; CCI: Canadian Classification of Interventions; DAD: Discharge Abstract Database; ICD-9: International Classification of Diseases, 9th Revision; ICD-10-CA: International Classification of Diseases, 10th Revision, Canada; IRCC: Immigration, Refugees and Citizenship Canada; NACRS: National Ambulatory Care Reporting System; ODD: Ontario Diabetes Dataset; OHIP: Ontario Health Insurance Plan; OLIS: Ontario Laboratories Information System; SDS: Same-Day Surgery Database

Assume below unless stated:

**CIHI-DAD**

Source

All

Institution types

Acute care (insttype = ‘AP’ or ‘AT’)

Include suspected/questionable diagnoses?

No

**OHIP**

Claim Type

NONLAB

**NACRS**

Source

Emergency Department visits

Include planned visits

No

**Supplementary Table 2.** Characteristics of the **preconception** exposure group and the **non-screened cohort** compared with standardized differences. All data shown are as a number (%) unless otherwise noted.

| **Characteristic** | **Preconception screened group (N = )** | **Non-screened cohort (N = )** | **Standardized difference** |
| --- | --- | --- | --- |
| *From 23 weeks’ gestation up to 42 days after the index delivery* |  |  |  |
| Total with SMM or death |  |  |  |
| Number of SMM indicators, median (IQR) |  |  |  |
| *At the time of A1c screening* |  |  |  |
| Mean maternal age, years |  |  |  |
| Maternal world region of origin |  |  |  |
| First |  |  |  |
| Second |  |  |  |
| Third |  |  |  |
| Fourth |  |  |  |
| Other |  |  |  |
| Multifetal pregnancy |  |  |  |
| Parity, median (IQR) |  |  |  |
| Nulliparity |  |  |  |
| Rural residence |  |  |  |
| Residence in the lowest income quintile area |  |  |  |
| Mean maternal A1c, % |  |  |  |
| Women < 5.8% A1c |  |  |  |
| Women 5.8%-6.4% A1c |  |  |  |
| Women > 6.4% A1c |  |  |  |
| Mean maternal pre-pregnancy BMI^a^, kg/m^2^ |  |  |  |
| Diabetes mellitus |  |  |  |
| *Conditions in year before index delivery* |  |  |  |
| Illegal drug or tobacco use |  |  |  |
| Chronic hypertension |  |  |  |

**Supplementary Table 3**. Further information on the relationship of severe maternal morbidity (SMM) risk factors to maternal glycemic control. For additional analysis 6, SMM risk factors were separated into those with likely, possible, or unlikely relationships to maternal glycemic control. The specific ICD-10-CA or CCI codes are identified, and where necessary, references are provided for support. For clarity, the SMM indicators have been grouped after reference 1.

| **Indicator Class** | **SMM Indicator** | **ICD-10-CA or CCI Codes** | **Reference** |
| --- | --- | --- | --- |
| *SPE, HELLP, Eclampsia* | Severe pre-eclampsia, HELLP syndrome | O14.1, or O14.2 | 2 |
|  | Eclampsia | O15 | 2 |
| *Severe Hemorrhage* | Placenta previa with hemorrhage and red cell transfusion | O44.1 + CIHI BTREDBC = 1 |  |
|  | Placental abruption with coagulation defect | O45.0 |  |
|  | Antepartum hemorrhage with coagulation defect | O46.0 | 15 |
|  | Intrapartum hemorrhage with coagulation defect | O67.0 | 15 |
|  | Intrapartum hemorrhage with red cell transfusion | O67 + CIHI BTREDBC = 1 | 15 |
|  | Postpartum hemorrhage with red cell transfusion, procedures to the uterus or hysterectomy | O72 + any of the following:   - BTREDBC = 1, or - (1.RM.13, 1.KT.51, 5.PC.91.LA or 5.PC.91.HV) + BTREDBC = 1, or - (5.MD.60.RC, 5.MD.60.RD, 5.MD.60.KE, 5.MD.60.CB or 1.RM.89.LA**^b^**), or - 1.RM.87.LA-GX   **^b^ NOTE**: **1.RM.89.LA** is included only if codes 1.PL.74, 1.RS.74 or 1.RS.80 are NOT also present | 15 |
|  | Curettage with red cell transfusion | (5.PC.91.GA, 5.PC.91.GC, 5.PC.91.GD) + CIHI BTREDBC = 1 |  |
| *Maternal ICU Admission* | Maternal ICU admission | SCU in (‘10’, ’20’, ’25’, ’30’, ’35’, ’40’,’45’,’60’,’80’) | 3 |
| *Surgical Complications* | Complications of obstetric surgery and procedures | O75.4 | 12 |
|  | Evacuation of incisional hematoma with RBC transfusion | 5.PC.73.JS + CIHI BTREDBC = 1 |  |
|  | Repair of bladder, urethra, or intestine | 5.PC.80.JR, 1.NK.80, 1.NM.80 |  |
|  | Reclosure of caesarean wound with RBC transfusion | (5.PC.80.JM, 5.PC.80.JH) + CIHI BTREDBC = 1 |  |
| *Hysterectomy* | Caesarean hysterectomy, hysterectomy using an open approach | 5.MD.60.RC, 5.MD.60.RD, 5.MD.60.KE, 5.MD.60.CB, 1.RM.89.LA (exclude if 1.PL.74, 1.RS.74 or 1.RS.80 code also present), 1.RM.87.LA-GX |  |
| *Sepsis* | Puerperal sepsis | O85 | 4, 5 |
|  | Septicemia during labour | O75.3 | 4, 5 |
| *Embolism, Shock, DIC* | Obstetric embolism | O88 | 14 |
|  | Obstetric shock | O75.1, R57, T80.5 or T88.6 |  |
|  | Disseminated intravascular coagulation | D65 | 7 |
| *Assisted Ventilation* | Assisted ventilation through endotracheal tube | 1.GZ.31.CA-ND |  |
|  | Assisted ventilation through tracheostomy | 1.GZ.31.CR-ND |  |
| *Cardiac Conditions* | Cardiomyopathy, cardiac arrest and resuscitation, myocardial infarction, pulmonary edema and heart failure | O74.2, O89.1, O90.3, I21, I22, I42, I43, I46, I49.0, I50, J81, 1.HZ.09 or 1.HZ.30 | 8, 9 |
| *Acute Renal Failure* | Acute renal failure | O90.4, N17, N19 or N99.0 | 6 |
|  | Dialysis | 1.PZ.21 | 6 |
| *Severe Uterine Rupture* | Rupture of the uterus with red cell transfusion,  procedures to the uterus or hysterectomy | (O71.0 or O71.1) + any of the following:   - CIHI BTREDBC = 1, or - (1.RM.13, 1.KT.51, 5.PC.91.LA or 5.PC.91.HV) + CIHI BTREDBC = 1, or - (5.MD.60.RC, 5.MD.60.RD, 5.MD.60.KE, 5.MD.60.CB or 1.RM.89.LA**^a^**), or - 1.RM.87.LA-GX   **^a^ NOTE**: **1.RM.89.LA** is included only if codes 1.PL.74, 1.RS.74 or 1.RS.80 are NOT also present | 13 |
| *Cerebrovascular Accidents* | Cerebral venous thrombosis in pregnancy | O22.5 | 14 |
|  | Cerebral venous thrombosis in the puerperium | O87.3 | 14 |
|  | Subarachnoid and intracranial hemorrhage, cerebral infarction | I60, I61, I62, I63, or I64 | 17 |
|  | Acute fatty liver with red cell transfusion or plasma  transfusion | O26.6 + (CIHI BTREDBC = 1 or CIHI BTPLASMA = 1) | 16 |
| *Miscellaneous* | Pulmonary, cardiac, and CNS complications of anesthesia during pregnancy, labour, delivery or the puerperium | O29.0, O29.1, O29.2, O89.0, O89.1, O89.2, O74.0, O74.1, O74.2 or O74.3 | 12 |
|  | Status asthmaticus | J45.01, J45.11, J45.81 or J45.91 | 10 |
|  | Adult respiratory distress syndrome | J80 | 3 |
|  | Hepatic failure | K71 or K72 | 18 |
|  | Acute abdomen | K35, K37, K65, N73.3 or N73.5 |  |
|  | Surgical or manual correction of inverted uterus for vaginal births only | 5.PC.91.HQ or 5.PC.91.HP, restricted to vaginal births (i.e., absence of caesarean 5.MD.60) |  |
|  | Sickle-cell anemia with crisis | D57.0 |  |
|  | Acute psychosis | F53.1 or F23 |  |
|  | Status epilepticus | G41 |  |
|  | HIV disease | B24 |  |

| Quality Assurance Activities | | | |
| --- | --- | --- | --- |
| **RAE Directory of SAS Programs** | U:/apark/projects/HSPE/p0990.169.000.RENAL/Obj4/ | | |
| **RAE Directory of Final Dataset(s)** | *The* *final analytic dataset for each cohort includes all the data required to create the baseline tables and run all the models. It should include all covariates for all models such as patient risk factors, hospital characteristics, physician characteristics, exposure measures (continuous, categorical) and outcomes. It should include covariates that were considered but didn’t make the final cut. This would permit an analyst to easily re-run the models in the future.* | | |
|  | /sasroot/projects/hspe/p0990.169.000/level1/apark/ | | |
| **RAE README file available:** Yes No | | | |
| **Date results of quality assurance tools for final dataset shared with project team (where applicable):** | | |  |
|  | | **%assign** | yyyy-mon-dd |
|  | | **%evolution** | yyyy-mon-dd |
|  | | **%dinexplore** | yyyy-mon-dd |
|  | | **%track / %exclude** | yyyy-mon-dd |
|  | | **%codebook** | yyyy-mon-dd |
| **Additional comments:**  Feasibility analysis for potential objective #4: Relation between HbA1c and SMM  - Calculate # pregnancies with any HbA1c test from -10 to +20^6/7^ wks of conception.  - Calculate % PTB.  There would be ~ 1798 SMM events (at a rate of 1.7%)  Among the 9569 pregnancies with HbA1C and ODD status, there would be xxx SMM events  The numbers are likely high enough for a multivariable fractional polynomial regression approach.  This would especially be so if we ran a model with HbA1C quintiles.  Or, a model, in which we examined the RR of SMM per 1 SD increase in HbA1c | |  | |
